# Compound 4′-O-methylbroussochalcone B attenuates LPS/ATP-induced macrophage pyroptosis in vitro through modulation of the NLRP3 inflammasome and MAPK/NF-κB pathways

**DOI:** 10.3389/fphar.2026.1807018

**Published:** 2026-06-24

**Authors:** Jihee Choi, You Chul Chung, Ami Lee, Han Na Kang, Youn-Hwan Hwang

**Affiliations:** 1 KM Convergence Research Division, Korea Institute of Oriental Medicine, Daejeon, Republic of Korea; 2 Korean Convergence Medical Science Major, KIOM School, University of Science & Technology (UST), Daejeon, Republic of Korea; 3 Research Center for Antibody platform and Infectious Disease, Scripps Korea Antibody Institute (SKAI), Hongcheon-gun, Republic of Korea

**Keywords:** 4′-O-methylbroussochalcone B, macrophage inflammation, MAPK/NF-κB signaling, mitochondrial ROS, NLRP3 inflammasome, pyroptosis

## Abstract

**Background:**

The compound 4′-O-methylbroussochalcone B (4′-O-M) is a chalcone-type flavonoid isolated from *Psoralea corylifolia*, a traditional Asian medicinal herb. While studies have reported its antioxidant and anti-inflammatory activities, its effects on macrophage inflammatory signaling and NLRP3 inflammasome activation remain unclear. Therefore, in this *in vitro* study, we aimed to clarify the anti-inflammatory actions and underlying molecular mechanisms of 4′-O-M in lipopolysaccharide (LPS)/adenosine triphosphate (ATP)-stimulated J774A.1 macrophages, with a particular emphasis on mitochondrial reactive oxygen species (mtROS)-dependent NLRP3 inflammasome activation and MAPK/NF-κB signaling pathways.

**Methods:**

J774A.1 macrophages were pretreated with graded concentrations of 4′-O-M, followed by LPS priming and ATP stimulation to activate the inflammasome. Levels of nitric oxide (NO), prostaglandin E_2_ (PGE_2_), and cytokines (interleukin [IL]-6, tumor necrosis factor-α [TNF-α], IL-1β, IL-18) in J774A.1 macrophages were quantified by ELISA. MAPK/NF-κB phosphorylation and inflammasome-related proteins (NLRP3, ASC, caspase-1, IL-1β, gasdermin D) were assessed by immunoblotting. mtROS production was evaluated using the MitoSOX™ Red assay.

**Results:**

4′-O-M markedly reduced LPS-induced inflammatory mediators, suppressing NO and PGE_2_ production and IL-6 and TNF-α secretion by inhibiting MAPK and NF-κB phosphorylation. As the response progressed to the inflammasome activation stage, 4′-O-M further reduced IL-1β and IL-18 release and limited caspase-1 activation. Additionally, 4′-O-M decreased the production of mtROS, an upstream signal associated with NLRP3 activation, suggesting that this reduction may contribute to its inhibitory effects on the inflammasome pathway.

**Conclusion:**

4′-O-M mitigates macrophage inflammatory responses *in vitro* via the dual suppression of MAPK/NF-κB-mediated priming and mtROS-dependent NLRP3 inflammasome activation. Thus, 4′-O-M is a promising natural scaffold for developing therapeutics targeting inflammasome-driven inflammatory diseases.

## Graphical Abstract

**Figure:**
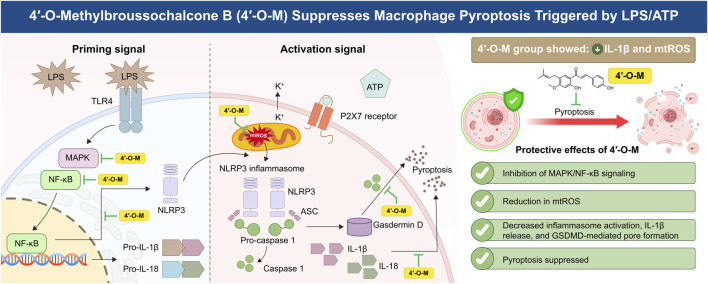
4′-O-Methylbroussochalcone B (4′-O-M) attenuates LPS/ATP-induced macrophage pyroptosis through dual-phase modulation of NLRP3 inflammasome signaling. At the priming stage, 4′-O-M inhibits Toll-like receptor 4 (TLR4)-driven MAPK and NF-κB activation, reducing transcription of pro-IL-1β, pro-IL-18, and NLRP3. At the activation stage, 4′-O-M decreases ATP-induced mitochondrial reactive oxygen species (mtROS) accumulation, limiting NLRP3 inflammasome assembly, caspase-1 cleavage, gasdermin D (GSDMD) processing, IL-1β/IL-18 maturation, and pyroptotic cell death.

## 1 Introduction

Inflammation is an essential biological process that enables the host to defend against infection and tissue injury (Kotas and Medzhitov, 2015). However, persistent or uncontrolled inflammation can lead to chronic inflammatory disorders such as arthritis, diabetes, neurodegenerative disorders, and cardiovascular diseases (Furman et al., 2019). Conventional anti-inflammatory agents, including corticosteroids and non-steroidal anti-inflammatory drugs, provide symptom relief but are often associated with adverse effects such as immunosuppression and organ toxicity (Dinarello, 2010; Rainsford, 2007). Consequently, interest in natural products as safer alternatives has significantly increased owing to their multi-targeted actions and biocompatible properties (Pan et al., 2010).

Phytochemicals from medicinal plants have gained increasing attention because they can modulate multiple pathways associated with inflammation, oxidative stress, and immune regulation (Atanasov et al., 2021). Unlike single-target synthetic drugs, these compounds often exert synergistic or pleiotropic effects, contributing to immune homeostasis with relatively low cytotoxicity. Among them, chalcones represent a notable class of flavonoids with antioxidant, antimicrobial, and anti-inflammatory activities (Thapa et al., 2023), and accumulating evidence suggests their therapeutic potential in chronic inflammatory and metabolic diseases (Adhikari et al., 2025).

*Psoralea corylifolia* (PC), a medicinal plant used in traditional Asian medicine, contains diverse bioactive constituents such as flavonoids, meroterpenes, and coumarins. Previous phytochemical investigations on PC identified 4′-O-methylbroussochalcone B (4′-O-M), a chalcone derivative, as one of its components and suggested that it has potential anti-inflammatory activity (Chen et al., 2023; Kim et al., 2010; Lee et al., 2021). Our preliminary screening indicated that 4′-O-M suppresses NF-κB reporter activity, suggesting that it may modulate inflammation-related signaling cascades (Kim et al., 2010). However, the precise molecular mechanisms by which 4′-O-M regulates inflammatory responses remain poorly understood. Specifically, whether 4′-O-M can modulate macrophage activation and inflammasome-mediated pyroptosis has not been elucidated.

Macrophages play a central role in innate immunity by producing pro-inflammatory cytokines and reactive oxygen species (ROS) upon activation by lipopolysaccharide (LPS) and adenosine triphosphate (ATP) (Kawai and Akira, 2010). Among various inflammasome complexes, the NOD-, LRR-, and pyrin domain-containing protein 3 (NLRP3) inflammasome is the most extensively studied owing to its broad responsiveness to bacterial toxins, ATP, crystalline particles, and metabolic stress signals (Paik et al., 2021). Activation of this complex represents a critical switch linking macrophage inflammation to pyroptotic cell death through caspase-1 activation and gasdermin D cleavage (Jiang et al., 2025). Additionally, mitochondrial ROS (mtROS) have emerged as central mediators connecting metabolic stress to NLRP3 inflammasome activation. Excessive ROS production amplifies inflammatory signaling and destabilizes mitochondrial membranes, further promoting inflammasome assembly (Zhou et al., 2011). Therefore, inhibition of mtROS generation is a promising strategy to attenuate inflammasome-driven inflammation and pyroptosis (Abderrazak et al., 2015; Guo et al., 2023). Natural flavonoids and chalcone derivatives reportedly modulate redox balance and inhibit inflammasome activation; however, the molecular basis of this regulation remains incompletely defined (Li et al., 2022). Specifically, whether 4′-O-M can modulate macrophage activation and inflammasome-mediated pyroptosis is unclear.

Based on these considerations, we hypothesized that 4′-O-M mitigates inflammation and pyroptosis via the modulation of mtROS-dependent NLRP3 activation and its upstream mitogen-activated protein kinase (MAPK)/nuclear factor kappa B (NF-κB) signaling. Accordingly, in this study, we evaluated the effects of 4′-O-M on LPS/ATP-stimulated J774A.1 macrophages by assessing nitric oxide (NO), prostaglandin E_2_ (PGE_2_), and cytokine secretion. Furthermore, we examined its influence on MAPK/NF-κB phosphorylation, NLRP3 inflammasome assembly, and pyroptotic execution. Additionally, ROS modulation experiments were performed to clarify the contribution of redox regulation. Finally, network pharmacology analysis and molecular docking simulation were conducted to identify potential protein targets involved in the anti-inflammatory actions of 4′-O-M (Singh et al., 2025). Thus, this study delineates the molecular mechanisms through which 4′-O-M suppresses macrophage inflammation, demonstrating that it attenuates LPS/ATP-induced pyroptosis by concurrently inhibiting MAPK/NF-κB signaling and mtROS-dependent activation of the NLRP3 inflammasome.

## 2 Materials and methods

### 2.1 Cell culture

J774A.1 murine macrophages were obtained from the American Type Culture Collection (ATCC, United States). The cells were cultured in Dulbecco’s Modified Eagle’s Medium (DMEM; Gibco, United States) supplemented with 10% fetal bovine serum (FBS; Gibco, United States), 100 U/mL penicillin, and 100 μg/mL streptomycin (Sigma-Aldrich, United States) incubated at 37 °C in a humidified 5% CO_2_ atmosphere. The cells were sub-cultured every 2–3 days to maintain exponential growth.

### 2.2 Preparation of 4′-O-Methylbroussochalcone B

4′-O-Methylbroussochalcone B (4′-O-M; molecular formula C_21_H_22_O_4_; molecular weight 338.4 g/mol; PubChem CID 321765; purity ≥98%) was commercially obtained from [ChemFaces Biotech Biotechnology Co., Ltd. (Wuhan, China), Cat. No. CFN98046] and used without further purification. The chemical identity and purity were verified by the manufacturer’s certificate of analysis based on HPLC-DAD and ^1^H-NMR analyses (provided in Supplementary Figure S2). Owing to its prenylated chalcone scaffold and high lipophilicity, 4′-O-M is practically insoluble in water and was therefore dissolved in dimethyl sulfoxide (DMSO) to prepare a 50 mM stock solution, which was aliquoted and stored at −20 °C protected from light. Working solutions were freshly prepared by serial dilution in culture medium immediately before each experiment, with the final DMSO concentration maintained at 0.1% (v/v) in all treatment wells. The vehicle control group consisted of complete culture medium containing 0.1% (v/v) DMSO; preliminary experiments confirmed that this vehicle concentration had no detectable effect on cell viability or any inflammatory readout compared with medium-only controls. The tested concentration range (3.125–50 µM) was selected based on (i) preliminary CCK-8 cytotoxicity screening establishing 25 µM as the maximum non-cytotoxic concentration in J774A.1 macrophages, (ii) bioactive concentrations of structurally related prenylated chalcones (e.g., broussochalcone A, licochalcone B) reported in the literature for macrophage anti-inflammatory studies (typically 5–25 µM), and (iii) achievable plasma concentrations of 4′-O-M predicted from a previous pharmacokinetic study of orally administered *P. corylifolia* extract (Lee et al., 2021).

### 2.3 Cell viability assay

Cell viability was determined using the Cell Counting Kit-8 (CCK-8; Dojindo Molecular Technologies, Cat# CK04-11) according to the manufacturer’s instructions. Briefly, J774A.1 cells were seeded in 96-well plates at a density of 5 × 10^4^ cells/well and incubated for 18 h at 37 °C. Cells were then treated with various concentrations of 4′-O-M (0, 3.125, 6.25, 12.5, 25, and 50 µM) for 24 h. Subsequently, 10 µL of CCK-8 reagent was added to the cells and incubated for an additional 2 h. Absorbance was recorded at 450 nm using a microplate reader (SpectraMax, Molecular Devices, United States). Cell viability was expressed as a percentage of the untreated control group. All experiments were performed in triplicate.

### 2.4 Cytokine measurement

Cells were seeded in 96-well plates at a density of 5 × 10^4^ cells/well and allowed to adhere overnight. The cells were then pretreated with 4′-O-M (12.5, 25, and 50 μM; dissolved in DMSO with final 0.1% v/v in all wells including vehicle controls) for 3 h, followed by stimulation with LPS (100 ng/mL) for 24 h. The culture supernatants were collected, and the levels of NO, prostaglandin E_2_ (PGE_2_), IL-1β, IL-6, and tumor necrosis factor-α (TNF-α) were quantified using commercial ELISA kits (eBioscience, San Diego, CA, United States) according to the manufacturer’s protocols.

### 2.5 Lactate dehydrogenase release assay

Cells were seeded in 96-well plates at a density of 5 × 10^4^ cells/well and incubated overnight. The cells were then primed with LPS (100 ng/mL) for 5.5 h, followed by treatment with 4′-O-M (12.5, 25, or 50 µM) for 1 h, and stimulation with ATP (2 mM) for 30 min to activate the inflammasome. The supernatants were collected, and lactate dehydrogenase (LDH) release was quantified using a colorimetric assay kit following the manufacturer’s instructions. Absorbance was measured at 450 nm using a microplate reader.

### 2.6 Western blotting

Total protein was extracted from cells using RIPA lysis buffer containing protease and phosphatase inhibitors (Thermo Scientific, United States). Equal amounts of the protein (10–20 μg, optimized per target) were separated by SDS–PAGE and transferred to nitrocellulose membranes (Bio-Rad, United States). Membranes were blocked with 5% skimmed milk for 1 h and incubated overnight at 4 °C with primary antibodies against caspase-1 (#83383), cleaved caspase-1 (#89332), ASC (#67824), NLRP3 (#15101), gasdermin D (GSDMD; #39754), IL-1β (#12242), cleaved IL-1β (#63124), phospho-ERK (#4376), ERK (#4696), phospho-p38 (#4511), p38 (#9212), phospho-IκBα (#2859), IκBα (#4812), NF-κB p65 (#8242), Lamin B1 (#12586), and β-actin (#3700) (all from Cell Signaling Technology, unless otherwise indicated). After washing, membranes were incubated with HRP-conjugated secondary antibodies and developed using enhanced chemiluminescence reagents (SuperSignal™ West Pico PLUS, Thermo Scientific, Cat# 34580). All experiments were conducted at least in triplicate (uncropped, full-length blot membranes are provided in Supplementary Figure S6).

### 2.7 Immunofluorescence staining

Cells were seeded in 96-well black clear-bottom plates (Corning, United States) and allowed to adhere overnight. Subsequently, the cells were stimulated with LPS (100 ng/mL) for 5.5 h, treated with 4′-O-M (12.5, 25, or 50 µM) for 1 h, and exposed to ATP (2 mM) for 30 min. Thereafter, the cells were incubated with propidium iodide (PI; P3566) for 5 min and counterstained with Hoechst 33,258 (ab228549, Abcam). Fluorescence images were captured using an ImageXpress Micro 4 High-Content Screening System (Molecular Devices, United States), and PI-positive cell ratios were quantified using the MetaXpress software under identical exposure settings.

### 2.8 mtROS detection and pharmacological modulation

mtROS were detected using MitoSOX™ Red staining (Invitrogen, United States). Cells were stimulated with LPS (100 ng/mL) for 5 h to induce priming, followed by treatment with 4′-O-M for 3 h and ATP stimulation (2 mM) for 30 min to activate the inflammasome. mitoTEMPO (25 µM) was used as an antioxidant, while antimycin A (5 µM) served as an mtROS inducer to verify redox dependency. Fluorescence intensity was measured using a microplate reader and normalized to viable cell count.

### 2.9 Network pharmacological analysis

Network pharmacology analysis was employed to explore the potential molecular mechanisms of 4′-O-M in inflammatory diseases. Predicted target proteins of 4′-O-M were retrieved from the SwissTargetPrediction database (http://www.swisstargetprediction.ch/) by selecting “*Homo sapiens*” and using the “show all” option without applying probability score thresholds. Inflammatory disease–associated genes were collected from the GeneCards database (https://www.genecards.org/) using the keyword “inflammatory disease.” Only genes with a GeneCards relevance score ≥4 were included. Overlapping genes between predicted targets and disease-related genes were used for downstream analyses. Functional enrichment of overlapping genes was conducted using the Enrichr platform (https://maayanlab.cloud/Enrichr/), focusing on WikiPathways, MSigDB Hallmark, and Gene Ontology (GO) Biological Process databases. The top 10 pathways from each library were selected according to adjusted p-values (Benjamini–Hochberg correction). Pathway–gene interaction networks were constructed and visualized using Cytoscape v3.10.3.

### 2.10 Molecular docking analysis

Molecular docking studies were conducted to predict the potential interactions between 4′-O-M and inflammation-related target proteins. Crystal structures of human p38α MAPK (PDB ID: 4DLI), ERK (PDB ID: 5AX3), IKKβ (PDB ID: 4KIK), NF-κB p65 (PDB ID: 8TQD), NLRP3 (PDB ID: 6NPY), and caspase-1 (PDB ID: 1RWX) were retrieved from the Protein Data Bank (https://www.rcsb.org/). Protein structures were prepared by removing water molecules and adding polar hydrogens using the Discovery Studio software (BIOVIA, Dassault Systèmes, v2021). The ligand structure of 4′-O-M was obtained from the PubChem database (CID: 321,765) and prepared with the Merck molecular force field94 (mmFF94) using OpenBabel. AutoDock Vina was run using PyRx 1.0, with the prepared ligand and protein datasets, and docking simulations were performed using a grid box centered on the active or reported binding sites of each protein, with dimensions set to 20 × 20 × 20 Å to encompass the binding pocket (Supplementary Table S1). The exhaustiveness was set to 8, and the default scoring function was applied. Binding affinities were recorded as the lowest predicted binding energy (kcal/mol). The top-ranked docking conformations were selected based on binding affinity and pose clustering. Protein–ligand interactions, including hydrogen bonds, hydrophobic contacts, and π–π stacking, were visualized using Discovery Studio Visualizer.

### 2.11 Statistical analysis

All statistical analyses were performed using GraphPad Prism 9.0 (GraphPad Software, United States). Prior to parametric testing, the normality of data distribution was assessed using the Shapiro–Wilk test, and homogeneity of variance was evaluated using the Brown–Forsythe test (Supplementary Table S4). As all datasets satisfied the assumptions of normality (p > 0.05) and equal variance (p > 0.05), one-way analysis of variance (ANOVA) followed by Dunnett’s *post hoc* test was applied for multiple group comparisons. Data are expressed as the mean ± standard deviation (SD) of three independent biological replicates (n = 3). Differences were considered statistically significant at p < 0.05; exact p-values are reported in the figure panels and in Supplementary Table S3.

## 3 Results

### 3.1 4′-O-M suppresses LPS-induced pro-inflammatory mediator production in macrophages

**FIGURE 1:**
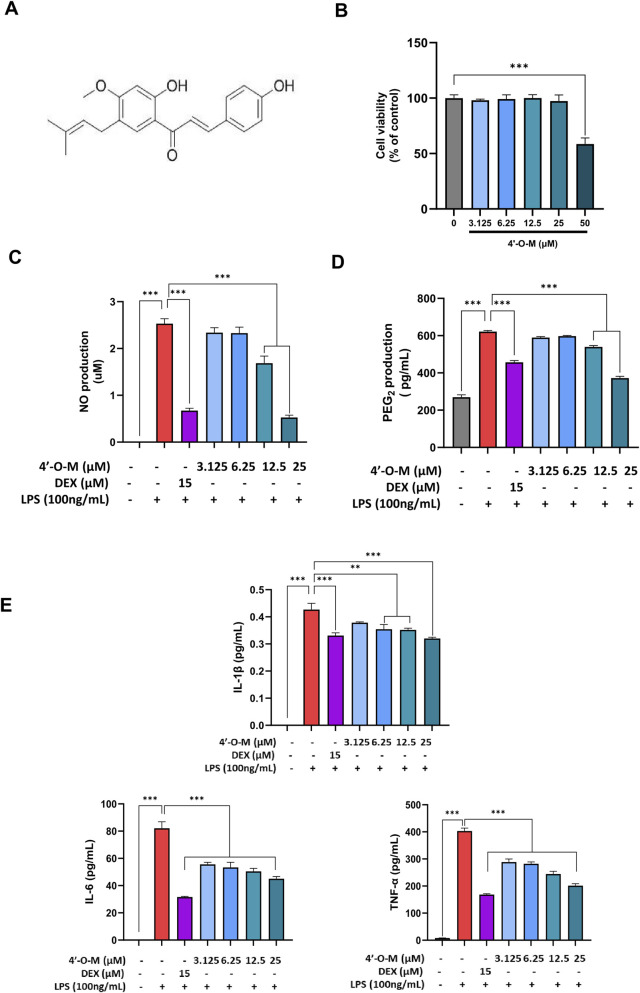
4′-O-M attenuates LPS-induced macrophage activation and inflammatory mediator production without inducing cytotoxicity J774A.1 macrophages were pretreated with the indicated concentrations (0, 3.125, 6.25, 12.5, 25, and 50 µM) of 4′-O-M for 1 h, followed by stimulation with LPS (100 ng/mL) for 24 h. Dexamethasone (DEX, 15 µM) served as a positive control. The vehicle control group consisted of complete medium containing 0.1% (v/v) DMSO. **(A)** Chemical structure of 4′-O-M. **(B)** Cell viability was determined by the CCK-8 assay. **(C,D)** Production of nitric oxide (NO) and prostaglandin E2 (PGE2) was quantified by Griess assay and ELISA, respectively. **(E)** Production of pro-inflammatory cytokines (IL-1β, IL-6, TNF-α) was measured by ELISA. Data are presented as the mean ± SD of three independent biological replicates (n = 3). Statistical significance among groups was determined by one-way ANOVA followed by Dunnett’s *post hoc* test, and is indicated as *p < 0.05, **p < 0.01, and ***p < 0.001 vs. the LPS-only group; exact p-values are provided in Supplementary Table S3. 4′-O-M, 4′-O-methylbroussochalcone B; CCK-8, Cell Counting Kit-8; DEX, dexamethasone; ELISA, enzyme-linked immunosorbent assay; IL, interleukin; LPS, lipopolysaccharide; NO, nitric oxide; PGE2, prostaglandin E2; SD, standard deviation; TNF-α, tumor necrosis factor-α.

We first evaluated the cytotoxicity of 4′-O-M in J774A.1 macrophages to assess its anti-inflammatory potential (Figure 1A). As shown in Figure 1B, 4′-O-M exhibited no detectable cytotoxicity up to 25 µM. However, a significant reduction in cell viability was observed at 50 µM (***p < 0.001 vs. vehicle control), indicating that concentrations ≤25 µM were appropriate for subsequent assays. Upon LPS stimulation (100 ng/mL, 24 h), macrophages produced large amounts of NO and PGE_2_, accompanied by the secretion of pro-inflammatory cytokines IL-1β, IL-6, and TNF-α. Conversely, pretreatment with 4′-O-M significantly decreased NO and PGE_2_ levels, as well as the production of cytokines in a concentration-dependent manner, comparable to the inhibitory pattern of dexamethasone (Figures 1C–E). These data indicate that 4′-O-M effectively attenuated LPS-induced macrophage activation and inflammatory mediator production without inducing cytotoxicity. Of note, IL-1β secretion under LPS stimulation alone (Figure 1E) showed a relatively flat dose–response curve across 3.125–25 μM, likely reflecting near-maximal suppression of NF-κB–dependent pro-IL-1β transcription at these concentrations. In contrast, mature IL-1β release under LPS/ATP stimulation (Figure 3B), which depends on both transcriptional priming and inflammasome-mediated processing, exhibited a clear concentration-dependent reduction by 4′-O-M, consistent with additional suppression at the activation step.

### 3.2 4′-O-M attenuates LPS-Induced inflammation by suppressing MAPK and NF-κB signaling pathways

**FIGURE 2:**
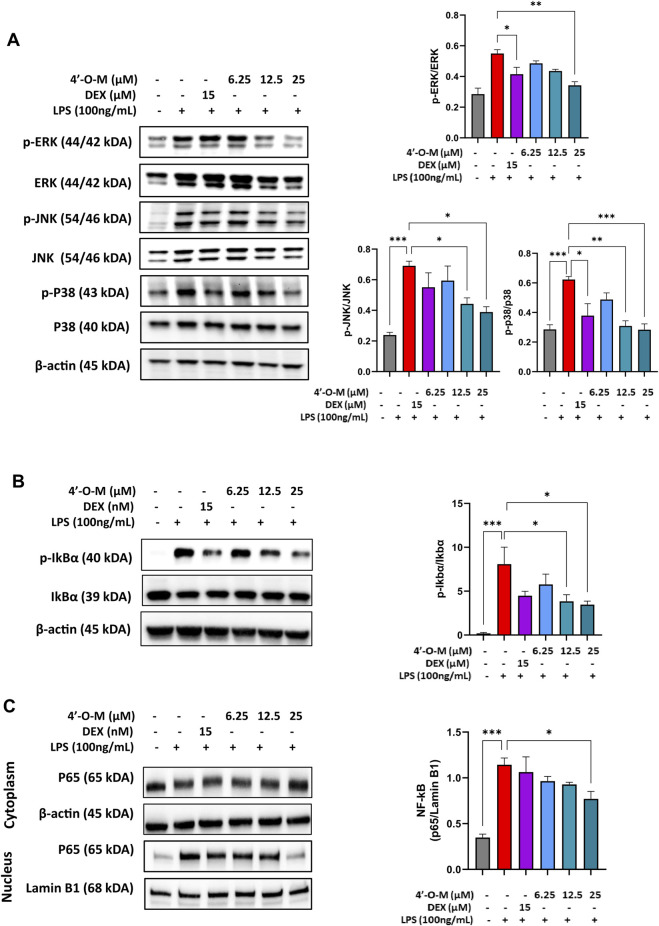
Effects of 4′-O-M on MAPK and NF-κB signaling pathways in LPS-stimulated J774A.1 macrophages. J774A.1 cells were pretreated with the indicated concentrations (6.25, 12.5, and 25 µM) of 4′-O-M for 1 h and then primed with LPS (100 ng/mL) for 2 h. Dexamethasone (DEX, 15 µM) served as a positive control; the vehicle control group consisted of complete medium containing 0.1% (v/v) DMSO. **(A)** Expressions of MAPK-related proteins (p-ERK and ERK, 44/42 kDa; p-JNK and JNK, 54/46 kDa; p-p38 and p38, 43 kDa; β-actin, 42 kDa) were analyzed by western blotting; densitometric quantification of phosphorylated/total protein ratios is shown on the right. **(B)** Expression and phosphorylation of IκBα (p-IκBα, 40 kDa; IκBα, 39 kDa; β-actin, 42 kDa) were examined to assess NF-κB activation. **(C)** Cytoplasmic and nuclear levels of NF-κB p65 (p65, 65 kDa; β-actin, 42 kDa for cytoplasmic loading; Lamin B1, 66 kDa for nuclear loading) were determined by western blotting. Data are presented as the mean ± SD of three independent biological replicates (n = 3); densitometric quantification was performed in ImageJ. Statistical analysis was performed by one-way ANOVA followed by Dunnett’s *post hoc* test (Shapiro–Wilk and Brown–Forsythe tests confirmed normality and homogeneity of variance, respectively). Significant differences are indicated as *p < 0.05, **p < 0.01, and ***p < 0.001 vs. the LPS-only group; exact p-values are provided in Supplementary Table S3. Uncropped, full-length blot membranes are presented in Supplementary Figures S4–S6. 4′-O-M, 4′-O-methylbroussochalcone B; DEX, dexamethasone; ERK, extracellular signal-regulated kinase; IκBα, inhibitor of κB alpha; JNK, c-Jun N-terminal kinase; Lamin B1, lamin B1 (nuclear envelope marker); LPS, lipopolysaccharide; MAPK, mitogen-activated protein kinase; NF-κB, nuclear factor kappa B; SD, standard deviation.

We examined the activation status of MAPK and NF-κB signaling components to investigate the mechanism underlying the anti-inflammatory effects of 4′-O-M. LPS stimulation markedly increased the phosphorylation of ERK1/2, JNK, and p38. In contrast, pretreatment with 4′-O-M significantly reduced the phosphorylation of all three MAPKs in a concentration-dependent manner (Figure 2A). Likewise, 4′-O-M suppressed the phosphorylation of IκBα and nuclear translocation of NF-κB p65, indicating prevention of IκBα degradation and NF-κB activation (Figures 2B,C). Collectively, these findings demonstrate that the anti-inflammatory effects of 4′-O-M are associated with concurrent suppression of MAPK and NF-κB signaling cascades.

### 3.3 4′-O-M suppresses NLRP3 inflammasome activation in LPS/ATP-stimulated macrophages

**FIGURE 3:**
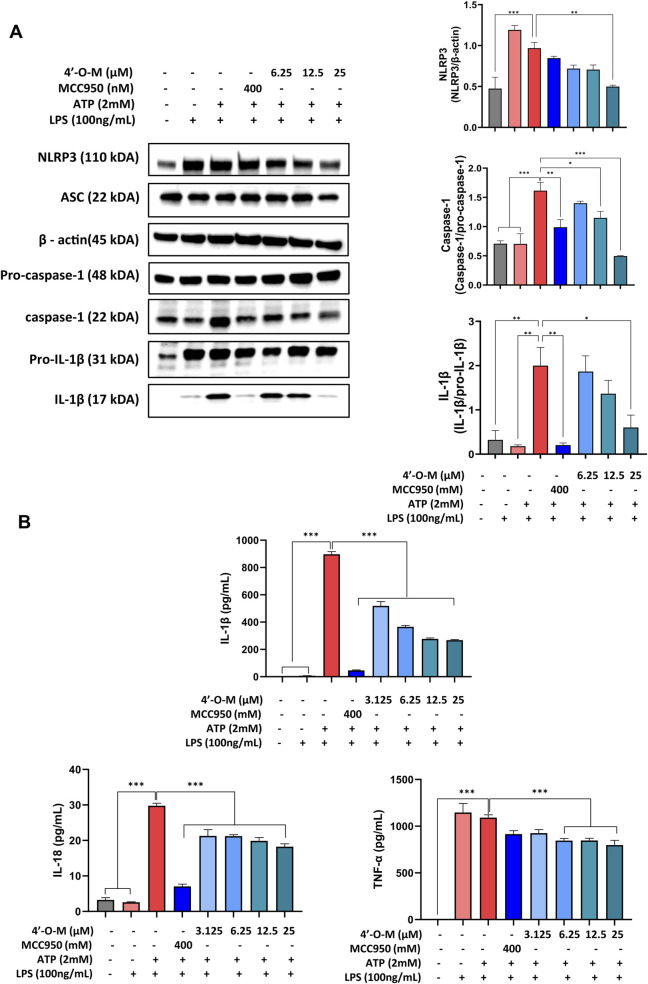
4′-O-M attenuates NLRP3 inflammasome activation in LPS/ATP-stimulated J774A.1 macrophages. J774A.1 cells were primed with LPS (100 ng/mL) for 5 h and treated with the indicated concentrations of 4′-O-M (6.25, 12.5, and 25 µM) for 1 h, followed by ATP (2 mM) stimulation for 30 min. MCC950 (400 nM) was used as a selective NLRP3 inflammasome inhibitor (positive control); the vehicle control group consisted of complete medium containing 0.1% (v/v) DMSO. **(A)** Expression of NLRP3 inflammasome–related proteins, including NLRP3 (110 kDa), ASC (22 kDa), pro-caspase-1 (45 kDa), cleaved caspase-1 p20 (20 kDa), pro-IL-1β (31 kDa), cleaved IL-1β (17 kDa), and β-actin (42 kDa), was analyzed by western blotting; densitometric quantification of NLRP3/β-actin, caspase-1/pro-caspase-1, and IL-1β/pro-IL-1β ratios is shown on the right. **(B)** Culture supernatants were collected and the secretion of IL-1β, IL-18, and TNF-α was quantified by ELISA. Data are presented as the mean ± SD of three independent biological replicates (n = 3); densitometric quantification was performed in ImageJ. Statistical analysis was performed by one-way ANOVA followed by Dunnett’s *post hoc* test (Shapiro–Wilk and Brown–Forsythe tests confirmed normality and homogeneity of variance, respectively). Significant differences are indicated as *p < 0.05, **p < 0.01, and ***p < 0.001 vs. the LPS/ATP-only group; exact p-values are provided in Supplementary Table S3. Uncropped, full-length blot membranes are presented in Supplementary Figures S4–S6. 4′-O-M, 4′-O-methylbroussochalcone B; ASC, apoptosis-associated speck-like protein containing a CARD; ATP, adenosine triphosphate; ELISA, enzyme-linked immunosorbent assay; IL, interleukin; LPS, lipopolysaccharide; MCC950, NLRP3-specific inhibitor; NLRP3, NOD-, LRR-, and pyrin domain–containing protein 3; SD, standard deviation; TNF-α, tumor necrosis factor-α.

Activation of the NLRP3 inflammasome is a key event linking inflammatory signaling to caspase-1–dependent pyroptosis. Therefore, we assessed inflammasome-related proteins in LPS/ATP-stimulated macrophages to determine whether 4′-O-M modulates this process. As shown in Figure 3A, LPS/ATP treatment markedly increased NLRP3 expression and promoted caspase-1 cleavage, which was accompanied by enhanced maturation of IL-1β. In contrast, total ASC protein levels remained largely unchanged, consistent with the fact that ASC is typically regulated through oligomerization rather than changes in overall expression. Treatment with 4′-O-M significantly reduced NLRP3 expression, caspase-1 activation, and the processing of pro-IL-1β, indicating that the compound interferes with inflammasome activation. Correspondingly, 4′-O-M lowered the secretion of IL-1β and IL-18 in a concentration-dependent manner and reduced LPS/ATP stimulation–induced TNF-α release (Figure 3B). These data suggest that 4′-O-M effectively attenuated NLRP3 inflammasome activation and downstream cytokine production.

### 3.4 4′-O-M inhibits LPS/ATP-induced pyroptosis in macrophages

**FIGURE 4:**
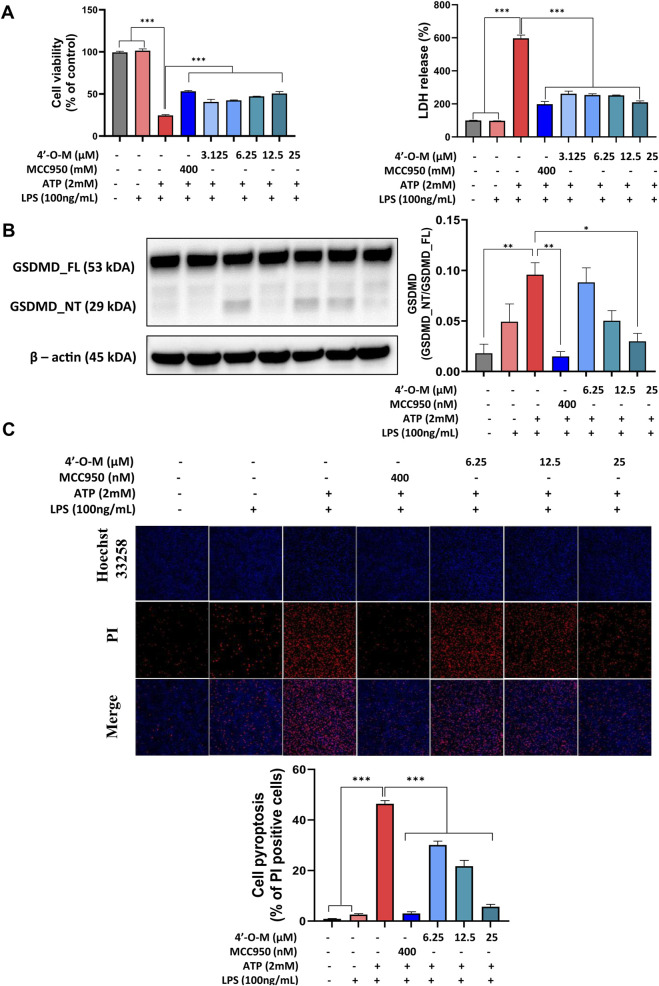
4′-O-M attenuates LPS/ATP-induced pyroptosis in J774A.1 macrophages. J774A.1 cells were primed with LPS (100 ng/mL) for 5 h, treated with the indicated concentrations of 4′-O-M (6.25, 12.5, and 25 µM) for 1 h, and then stimulated with ATP (2 mM) for 30 min. MCC950 (400 nM) was used as a selective NLRP3 inflammasome inhibitor (positive control); the vehicle control group consisted of complete medium containing 0.1% (v/v) DMSO. **(A)** Cell viability was measured by the CCK-8 assay, and cytotoxicity was measured by the lactate dehydrogenase (LDH) release assay. **(B)** Western blot analysis of gasdermin D (GSDMD-FL, 53 kDa) and its cleaved N-terminal fragment (GSDMD-NT, 31 kDa; β-actin, 42 kDa); densitometric quantification of the GSDMD-NT/GSDMD-FL ratio is shown on the right. **(C)** Hoechst 33,258 and PI double staining images were captured by fluorescence microscopy (20×), merged with bright-field images; the percentage of PI-positive (pyroptotic) cells is quantified in the bar graph below. Data are presented as the mean ± SD of three independent biological replicates (n = 3). Statistical analysis was performed by one-way ANOVA followed byDunnett’s *post hoc* test (Shapiro–Wilk and Brown–Forsythe tests confirmed normality and homogeneity of variance, respectively). Significant differences are indicated as *p < 0.05, **p < 0.01, and ***p < 0.001 vs. the LPS/ATP-only group; exact p-values are provided in Supplementary Table S3. Uncropped, full-length blot membranes are presented in Supplementary Figures S4–S6. 4′-O-M, 4′-O-methylbroussochalcone B; ATP, adenosine triphosphate; CCK-8, Cell Counting Kit-8; DMSO, dimethyl sulfoxide; GSDMD, gasdermin D; GSDMD-FL, full-length gasdermin D; GSDMD-NT, gasdermin D N-terminal fragment; LDH, lactate dehydrogenase; LPS, lipopolysaccharide; MCC950, NLRP3-specific inhibitor; PI, propidium iodide; SD, standard deviation.

LPS/ATP stimulation markedly decreased macrophage viability and elevated LDH release, consistent with pyroptotic membrane damage (Figure 4A). Notably, treatment with 4′-O-M restored cell viability and significantly reduced LDH release in a concentration-dependent manner, showing an inhibitory pattern similar to that of the NLRP3 inhibitor MCC950. At the molecular level, western blot analysis demonstrated that LPS/ATP robustly induced cleavage of GSDMD, generating the active N-terminal fragment responsible for membrane pore formation. This cleavage was markedly suppressed by 4′-O-M in a concentration-dependent manner (Figure 4B). Consistent with this molecular finding, fluorescence imaging further supported the protective effect of 4′-O-M against pyroptotic membrane damage: PI/Hoechst staining revealed abundant PI-positive cells following LPS/ATP exposure, whereas treatment with 4′-O-M substantially lowered the proportion of PI-positive cells (Figure 4C). Together, these results indicate that 4′-O-M effectively protected macrophages from inflammasome-dependent pyroptosis by limiting GSDMD activation and pyroptotic membrane disruption.

### 3.5 4′-O-M reduces mtROS levels in LPS/ATP-stimulated macrophages

**FIGURE 5:**
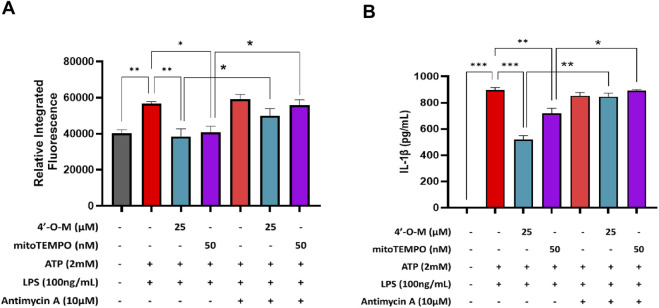
4′-O-M suppresses mitochondrial ROS production in LPS/ATP-stimulated J774A.1 macrophages J774A.1 cells were primed with LPS (100 ng/mL) for 5 h and pretreated with 4′-O-M (25 µM) for 3 h, followed by ATP (2 mM) stimulation for 30 min mitoTEMPO (50 nM) was used as a mitochondria-targeted antioxidant control, and antimycin A (10 µM) as a mitochondrial complex III inhibitor to elevate mtROS. The vehicle control group consisted of complete medium containing 0.1% (v/v) DMSO. **(A)** Mitochondrial ROS levels were quantified using the MitoSOX™ Red assay and expressed as relative integrated fluorescence intensity normalized to viable cell count. **(B)** IL-1β secretion in culture supernatants was measured by ELISA under the same treatment conditions. Data are presented as the mean ± SD of three independent biological replicates (n = 3). Statistical analysis was performed by one-way ANOVA followed by Dunnett’s *post hoc* test (Shapiro–Wilk and Brown–Forsythe tests confirmed normality and homogeneity of variance, respectively). Significant differences are indicated as *p < 0.05, **p < 0.01, and ***p < 0.001; exact p-values are provided in Supplementary Table S3. 4′-O-methylbroussochalcone B (4′-O-M); antimycin A, mitochondrial complex III inhibitor; ATP, adenosine triphosphate; DMSO, dimethyl sulfoxide; ELISA, enzyme-linked immunosorbent assay; IL, interleukin; LPS, lipopolysaccharide; mitoTEMPO, mitochondria-targeted antioxidant; mtROS, mitochondrial reactive oxygen species; ROS, reactive oxygen species; SD, standard deviation.

We monitored mtROS using the MitoSOX Red probe to evaluate whether mitochondrial redox changes contribute to the actions of 4′-O-M. As shown in Figure 5A, ATP markedly elevated mtROS signals in LPS-primed macrophages, however, pretreatment with 4′-O-M noticeably blunted this rise, with a degree of reduction similar to that achieved with mitoTEMPO, a reference mtROS scavenger. These observations indicate that 4′-O-M helps restrain the accumulation of mtROS under inflammasome-activating conditions. Subsequently, mtROS levels were increased upon treatment with antimycin A, a complex III inhibitor known to drive mtROS production, together with LPS/ATP (Figure 5A). A comparable pattern was reflected in IL-1β secretion (Figure 5B). Overall, these findings show that 4′-O-M diminishes mtROS generation and consequently reduces IL-1β release in macrophages undergoing NLRP3 inflammasome activation.

### 3.6 Network pharmacology overview of pathways potentially modulated by 4′-O-M, including MAPK–NF-κB-ROS signaling

**FIGURE 6:**
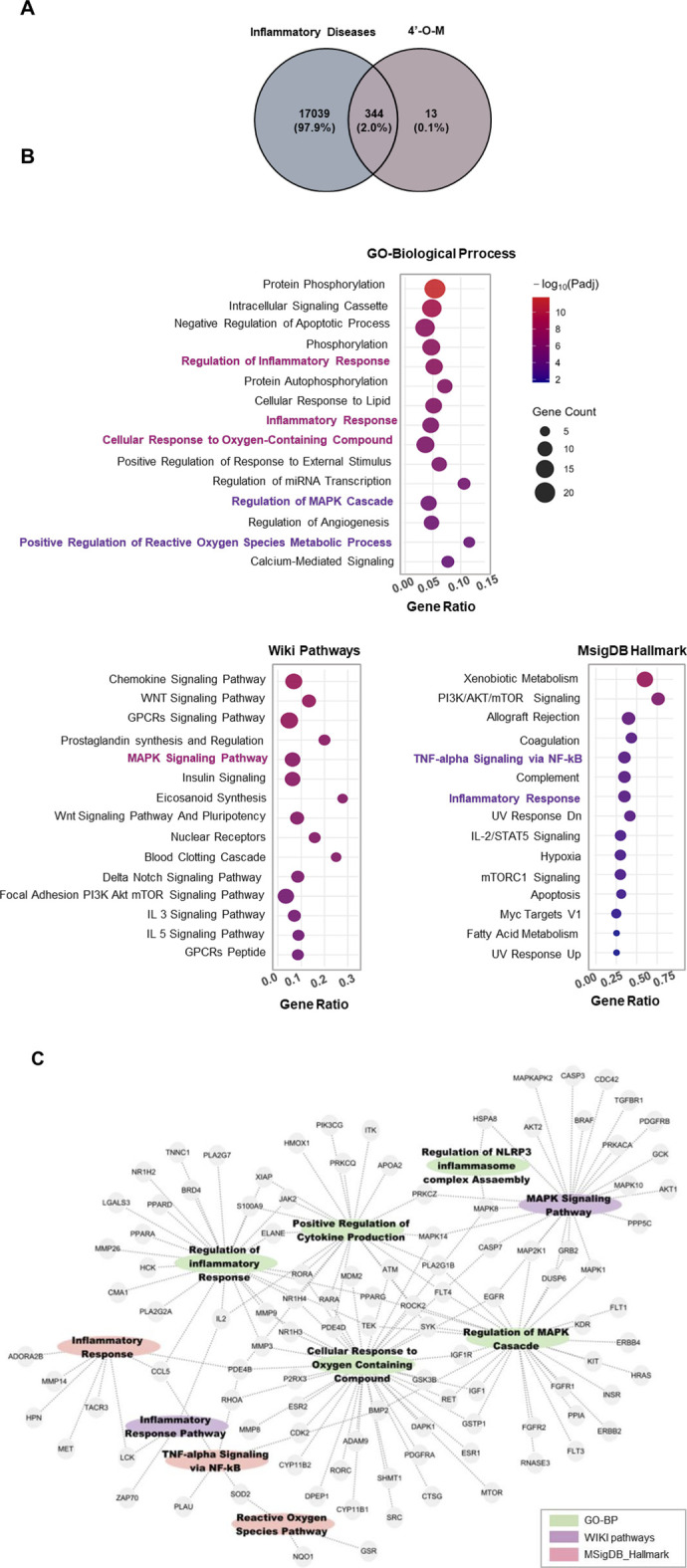
Network pharmacology analysis identifies MAPK/NF-κB–ROS signaling as major inflammatory hubs targeted by 4′-O-M **(A)** Venn diagram showing overlap between predicted human targets of 4′-O-M (n = 357 predicted targets retrieved from SwissTargetPrediction) and inflammatory disease–associated genes (GeneCards relevance score ≥4), identifying 344 common targets. **(B)** Bubble plots of pathway enrichment analysis results from GO-Biological Process, WikiPathways, and MSigDB Hallmark databases (Enrichr platform). Pathways are ranked by adjusted p-value (Benjamini–Hochberg correction); bubble size represents the number of overlapping genes, and color indicates significance (−log10 Padj). Top enriched pathways included regulation of inflammatory response, MAPK signaling pathway, and TNF-α signaling via NF-κB, with additional enrichment of ROS-related processes. **(C)** Pathway–gene interaction network constructed from significantly enriched terms using Cytoscape v3.10.3. Nodes represent pathways (colored by database) or genes (gray), and edges indicate pathway–gene associations. Hub genes MAPK1, MAPK14, RELA, NFKB1, NLRP3, and TNF emerged as central connectors, linking inflammatory signaling with ROS regulation. These hubs highlight the MAPK/NF-κB–ROS axis as a major mechanism by which 4′-O-M may exert anti-inflammatory effects. 4′-O-M, 4′-O-methylbroussochalcone B; GO, Gene Ontology; MAPK, mitogen-activated protein kinase; MAPK1, mitogen-activated protein kinase 1 (ERK2); MAPK14, mitogen-activated protein kinase 14 (p38α); NF-κB, nuclear factor kappa B; NFKB1, nuclear factor kappa B subunit 1; NLRP3, NOD-, LRR-, and pyrin domain–containing protein 3; RELA, v-rel avian reticuloendotheliosis viral oncogene homolog A (p65 subunit of NF-κB); ROS, reactive oxygen species; TNF-α, tumor necrosis factor-α.

A network pharmacology analysis was performed to further elucidate the molecular landscape underlying the anti-inflammatory actions of 4′-O-M. Among the predicted human targets of 4′-O-M, 344 genes overlapped with inflammation-related targets (Figure 6A), indicating substantial functional convergence between 4′-O-M activity and inflammatory disease pathways. Enrichment analysis of these overlapping genes revealed significant associations across GO-Biological Process, WikiPathways, and MSigDB Hallmark databases (Figure 6B). Prominent pathways included regulation of the inflammatory response, cellular response to oxygen-containing compounds, regulation of the MAPK cascade, MAPK signaling, and TNF-α signaling via NF-κB. Notably, several mtROS-related biological processes were also enriched, underscoring the interplay between oxidative stress regulation and inflammatory signaling. Therefore, a gene–pathway network was constructed using the top enriched terms to visualize these interactions (Figure 6C). The resulting network demonstrated clear clustering around inflammatory regulation, MAPK and NF-κB signaling modules, and pathways related to mitochondrial oxidative stress. Hub genes were predominantly composed of MAPK subfamilies and NF-κB regulatory proteins, highlighting their central role in linking immune activation with redox-dependent mechanisms. Together, these findings suggest that 4′-O-M exerts its anti-inflammatory effects by engaging interconnected MAPK, NF-κB, and mtROS-associated pathways, which emerge as key regulatory hubs within the predicted mechanism of action of 4′-O-M.

### 3.7 Molecular docking analysis predicts direct interactions between 4′-O-m and inflammation-related target proteins

Molecular docking simulations were performed against representative proteins involved in MAPK/NF-κB signaling and NLRP3 inflammasome activation to further clarify the molecular basis of 4′-O-M activity. Docking was conducted using AutoDock Vina, and the predicted binding poses were visualized with Discovery Studio Visualizer. As summarized in Supplementary Tables S2, S4 ′-O-M exhibited favorable binding affinities toward several inflammation-related proteins, including p38α MAPK (−9.6 kcal/mol), IKKβ (−9.7 kcal/mol), NLRP3 (−8.7 kcal/mol), and caspase-1 (−7.8 kcal/mol). Among them, IKKβ and p38α MAPK showed the strongest predicted affinities, suggesting that these kinases may represent primary interaction sites through which 4′-O-M modulates upstream inflammatory signaling. Further analysis of the docking poses showed that 4′-O-M formed several hydrogen bonds and numerous hydrophobic contacts within the ligand-binding regions of p38α MAPK and IKKβ, suggesting favorable accommodation in their regulatory pockets. In addition, π–π stacking and alkyl-type interactions were detected with key residues of NF-κB p65 and caspase-1, indicating additional stabilizing forces across multiple inflammatory targets. While molecular docking alone cannot establish direct enzymatic inhibition, these interaction patterns support the possibility that 4′-O-M engages several nodes within the MAPK and NF-κB signaling pathways and may also interact with components involved in NLRP3 inflammasome activation. Such multi-target binding characteristics are consistent with the compound’s broad anti-inflammatory profile. Collectively, these results suggest that 4′-O-M has the potential to engage multiple components of inflammatory signaling cascades, providing structural insight into its broad anti-inflammatory effects.

## 4 Discussion

Inflammation represents a tightly orchestrated host defense system that protects tissues from microbial invasion and injury. However, an excessive or unresolved inflammatory response can drive various chronic disorders, including autoimmune, metabolic, and neurodegenerative diseases (Kotas and Medzhitov, 2015). Macrophages serve as central coordinators of this process, integrating microbial and endogenous danger cues through pattern-recognition receptors. Among them, the NLRP3 inflammasome acts as a key molecular hub linking innate sensing to the maturation of cytokines and the execution of pyroptotic cell death (Paik et al., 2021). Consequently, pharmacological modulation of inflammasome priming and activation has gained recognition as an attractive therapeutic approach (Zhan et al., 2023).

In this study, we demonstrated that 4′-O-M, a chalcone derivative (Figure 1A) present in PC (Supplementary Table S1; Supplementary Figure S4), acted on the priming and activation stages of inflammasome signaling. During priming, LPS engages TLR4 to activate NF-κB and MAPK pathways, driving transcription of pro-IL-1β, pro-IL-18, and NLRP3 (Kawai and Akira, 2010). Treatment with 4′-O-M decreased ERK, JNK, and p38 phosphorylation, prevented IκBα degradation, and limited nuclear translocation of NF-κB p65 (Zheng et al., 2024), consistent with suppression of these upstream signals. Importantly, in our experimental design (Sections 2.5, 2.7, and 2.8), 4′-O-M was administered after LPS priming had been completed and immediately before ATP stimulation. Under this post-priming schedule, 4′-O-M still robustly suppressed GSDMD cleavage, IL-1β/IL-18 secretion, LDH release, and PI uptake, indicating that its anti-pyroptotic activity cannot be attributed solely to the inhibition of NF-κB–dependent transcriptional priming (Figures 3, 4). Intriguingly, the cytokine suppression profiles differed markedly depending on the timing of 4′-O-M administration: under LPS-only conditions where 4′-O-M was administered before LPS (Figure 1E), TNF-α was preferentially suppressed while IL-1β showed only modest reduction—consistent with predominant NF-κB inhibition at the priming step (Pan et al., 2010), (Atanasov et al., 2021). In contrast, under LPS/ATP conditions where 4′-O-M was administered after LPS priming (Figure 3B), IL-1β was substantially and concentration-dependently suppressed while TNF-α showed only modest reduction—consistent with predominant inhibition at the inflammasome activation step (Zhou et al., 2011), (Abderrazak et al., 2015). This timing-dependent shift in cytokine suppression selectivity, in which the same compound produces distinct suppression profiles depending solely on the timing of administration, supports a dual-action model in which 4′-O-M independently engages both the NF-κB–dependent priming step and the inflammasome activation step. We acknowledge, however, that this evidence is pharmacological rather than genetic; the involvement of MAPK and NF-κB signaling is therefore presented as an association rather than a definitively established causal mechanism, and is further addressed in the Limitations section.

Notably, our data indicate that these inhibitory effects occur without measurable cytotoxicity, reinforcing the notion that 4′-O-M selectively targets inflammatory signaling rather than compromising cell viability (Figure 2A). Additionally, the inhibitory profile of 4′-O-M was comparable to that of the well-established NLRP3 inhibitor Licochalcone B (Li et al., 2022), underscoring its potential for therapeutic application. Similarly, cardamonin, another naturally occurring chalcone sharing the α,β-unsaturated carbonyl scaffold was recently identified as a broad-spectrum NLRP3 inflammasome inhibitor that blocks ASC oligomerization and IL-1β release (Wang et al., 2019), underscoring the translational relevance of chalcone scaffolds in inflammasome modulation.

Beyond priming, we examined how 4′-O-M influences the activation phase. NLRP3 assembly is triggered by danger signals including mitochondrial dysfunction and ROS overproduction (Zhou et al., 2011; Abderrazak et al., 2015; Jaara and Torres, 2024) with ROS amplifying inflammasome activity both directly and through redox-sensitive transcription (Ziehr and MacDonald, 2024). Consistent with this, 4′-O-M reduced cytosolic and mitochondrial ROS in LPS/ATP-stimulated macrophages, accompanied by decreased mature IL-1β and IL-18 (Figure 3B). The mtROS dependency was further supported by antimycin A, a mitochondrial complex III inhibitor that elevates mtROS and promotes NLRP3 activation (Quinlan et al., 2011); 4′-O-M attenuated this response. These data position 4′-O-M as a redox-sensitive modulator that disrupts the ROS–NLRP3 axis to prevent pyroptotic cell death (Zhou et al., 2011; Abderrazak et al., 2015).

Network pharmacology analysis provided systems-level support for the experimental findings. The enrichment of MAPK signaling, TNF-α signaling via NF-κB, and ROS-related biological processes among the 344 overlapping targets of 4′-O-M (Figures 6A,B) is consistent with the observed suppression of ERK/JNK/p38 phosphorylation, IκBα degradation, and mtROS accumulation in LPS/ATP-stimulated macrophages. Furthermore, the identification of NLRP3 inflammasome complex assembly as a central hub in the protein–protein interaction network (Figure 6C) provides *in silico* corroboration for the dual-action mechanism identified experimentally. Collectively, these network-level findings reinforce the interpretation that 4′-O-M engages interconnected MAPK, NF-κB, and mtROS-associated pathways as key regulatory nodes underlying its anti-inflammatory activity.

**FIGURE 7:**
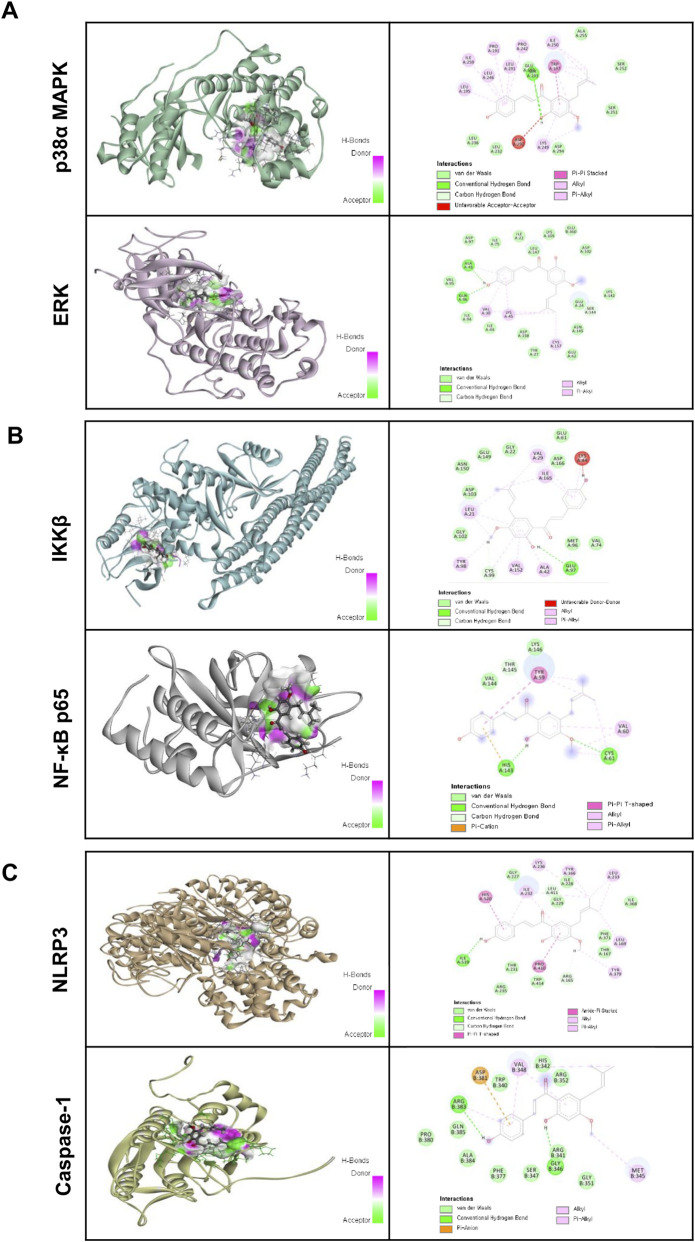
Molecular docking analysis of 4′-O-M with inflammation-related target proteins. Representative 3D and 2D docking interaction diagrams of 4′-O-M bound to **(A)** p38α MAPK (PDB ID: 4DLI) and ERK (PDB ID: 5AX3), **(B)** IKKβ (PDB ID: 4KIK) and NF-κB p65 (PDB ID: 8TQD), and **(C)** NLRP3 (PDB ID: 6NPY) and caspase-1 (PDB ID: 1RWX). The 3D models illustrate the predicted binding pocket, hydrogen-bond interactions, and hydrophobic contacts, whereas the corresponding 2D interaction maps highlight key residues involved in ligand stabilization. Predicted binding affinities (kcal/mol) for each protein are listed in Supplementary Table S2. Docking simulations were conducted using AutoDock Vina (via PyRx 1.0), and interaction diagrams were generated using Discovery Studio Visualizer v2021. Of note, molecular docking predicts geometric compatibility between ligand and protein and does not establish direct enzymatic inhibition; functional validation through biochemical or genetic approaches will be required to confirm these interactions. 4′-O-M, 4′-O-methylbroussochalcone B; caspase-1, cysteine-aspartic acid protease 1; ERK, extracellular signal-regulated kinase; IKKβ, inhibitor of κB kinase beta; MAPK, mitogen-activated protein kinase; NF-κB, nuclear factor kappa B; NLRP3, NOD-, LRR-, and pyrin domain–containing protein 3; PDB, Protein Data Bank; 2D, two-dimensional; 3D, three-dimensional.

Beyond direct ROS scavenging, 4′-O-M may engage upstream redox sensors. Chalcones are electrophilic and can modify cysteine residues on Keap1, stabilizing Nrf2 and inducing cytoprotective genes such as HO-1 and NQO1 (Cuadrado et al., 2019), (Bellezza et al., 2018); Nrf2 activation also suppresses thioredoxin-interacting protein (TXNIP), attenuating NLRP3 assembly (An et al., 2019). A second possibility involves the NIMA-related kinase 7 (NEK7) –NLRP3 interface, a structural checkpoint downstream of potassium efflux (Li et al., 2022), (Sharif et al., 2019). Covalent NEK7 inhibition disrupts this interaction and blocks inflammasome activation (Jin et al., 2024), (Broz and Dixit, 2016), and our docking analysis placed 4′-O-M within the NACHT domain near NEK7-binding residues (Figure 7), suggesting that steric or allosteric hindrance at this interface may contribute to its activity (He et al., 2016), (Zhang et al., 2025). Whether 4′-O-M acts through either of these mechanisms requires direct experimental validation.

The suppression of macrophage pyroptosis by 4′-O-M has potential immunological consequences. Pyroptotic death releases alarmins and damage-associated molecular patterns (DAMPs) that amplify tissue injury, so restraining this process can reduce pathological inflammation without abolishing microbial clearance (Karki and Kanneganti, 2019). Excessive IL-1β similarly drives metabolic inflammation and fibrosis, both of which respond to inflammasome targeting (Abderrazak et al., 2015). Our data show that 4′-O-M acts at the final execution step of canonical pyroptosis by suppressing GSDMD cleavage (Shi et al., 2015), (Orning et al., 2019), identifying GSDMD as a key downstream target relevant to its anti-pyroptotic activity.

Although the present study focused on the downstream convergence of MAPK/NF-κB priming and mtROS-dependent activation, the P2X7 receptor pathway warrants investigation as an important upstream node. Extracellular ATP binds P2X7 on macrophages, triggering K^+^ efflux—one of the best-characterized signals for NLRP3 activation. Whether 4′-O-M directly affects P2X7 channel function, intracellular K^+^ depletion, or downstream Ca^2+^ signaling remains to be determined. P2X7-selective antagonists (e.g., A438079, AZD9056), high-K^+^ buffers, and Ca^2+^ imaging would help define whether 4′-O-M acts at or upstream of this axis, and would strengthen the mechanistic framework established here.

Structurally, 4′-O-M sits within the chalcone family in a way that may underlie its activity. The 4′-O-methyl substitution and prenyl side chain likely enhance membrane permeability, increase lipophilicity, and improve both kinase-binding stability and radical-scavenging capacity (Martins et al., 2021), (Cheng et al., 2001), properties consistent with the observed ROS and MAPK inhibition. Rational modification of the phenolic hydroxyls or prenyl groups could further tune bioavailability and target selectivity in future drug design efforts (Cheng et al., 2001), (Liu et al., 2021).

Several limitations should be acknowledged. The findings are derived from a single *in vitro* macrophage model (J774A.1) and do not capture the cellular heterogeneity, tissue context, systemic feedback, or pharmacokinetics that govern inflammasome biology *in vivo*. Validation across additional macrophage models such as RAW 264.7, BMDMs, and human THP-1, as well as *in vivo* disease models including DSS colitis, LPS sepsis, atherosclerosis, and neuroinflammation, will be necessary to establish translational relevance (Jiang et al., 2025), (Liu et al., 2025). Although pharmacological evidence (post-priming treatment, mtROS rescue, and MCC950-comparable activity) supports dual action of 4′-O-M, genetic loss-of-function approaches such as NLRP3^−/−^, GSDMD^−/−^, NEK7 knockdown, and silencing of MAPK isoforms or IKKβ, together with rescue experiments, ASC oligomerization assays, and direct binding measurements (e.g., SPR or ITC), will be needed to confirm the causal hierarchy and the precise molecular target. Prior pharmacokinetic work indicates that 4′-O-M can reach bioactive systemic concentrations after oral administration (Kim et al., 2010), (Lee et al., 2021), supporting its translational potential as a chalcone-based scaffold (Thapa et al., 2023); structure–activity studies and pharmacokinetic profiling of chalcone derivatives would be valuable next steps.

In conclusion, our findings demonstrate that 4′-O-M attenuates NLRP3 inflammasome activity at both the priming and activation stages in association with modulation of MAPK/NF-κB signaling and reduction of mtROS accumulation, without inducing cytotoxicity at the tested concentrations. These findings should be regarded as a preliminary mechanistic foundation, and subsequent *in vivo* validation together with genetic functional studies is warranted before therapeutic implications can be drawn. Nevertheless, these mechanistic features position 4′-O-M as a promising multi-target candidate for disorders driven by inflammasome activation.

## Data Availability

The original contributions presented in the study are included in the article/Supplementary Material, further inquiries can be directed to the corresponding authors.
