# Supplementary material for: Compound 4′-O-methylbroussochalcone B attenuates LPS/ATP-induced macrophage pyroptosis in vitro through modulation of the NLRP3 inflammasome and MAPK/NF-κB pathways
Source: Front Pharmacol. 2026 Jun 24;17:1807018. doi: 10.3389/fphar.2026.1807018 (PMC13341802; doi:10.3389/fphar.2026.1807018)

Supplementary Material

# Supplementary Methods

## Ultrahigh-Performance Liquid Chromatography/Quadrupole-Orbitrap Mass Spectrometry (UHPLC–MS/MS)

*Psoralea corylifolia L*. seeds were purchased from a herbal Good Manufacturing Practice (hGMP) manufacturer (Gwangmyeongdang Pharm, Ulsan, Republic of Korea). The dried seeds (1 kg) were extracted with either distilled water or 70% ethanol (v/v) under reflux at the boiling point for 3 h. The extracts were then filtered and lyophilized.

Phytochemical profiling of PC was conducted using a Dionex UltiMate 3000 UHPLC system coupled to a Thermo Q-Exactive quadrupole-Orbitrap mass spectrometer (Thermo Fisher Scientific, CA, USA). Chromatographic separation was achieved using a Waters Acquity UPLC BEH C18 column (2.1 × 100 mm, 1.7 µm). The mobile phase consisted of 0.1% formic acid in water (A) and acetonitrile (B), and gradient elution conditions were applied according to a previous report. Samples were dissolved in methanol and filtered through a 0.22 µm membrane prior to injection. Phytochemical constituents were identified by comparing their retention times and MS/MS fragmentation patterns with those of authentic standards.

# Supplementary Figures and Tables

## Supplementary Figures


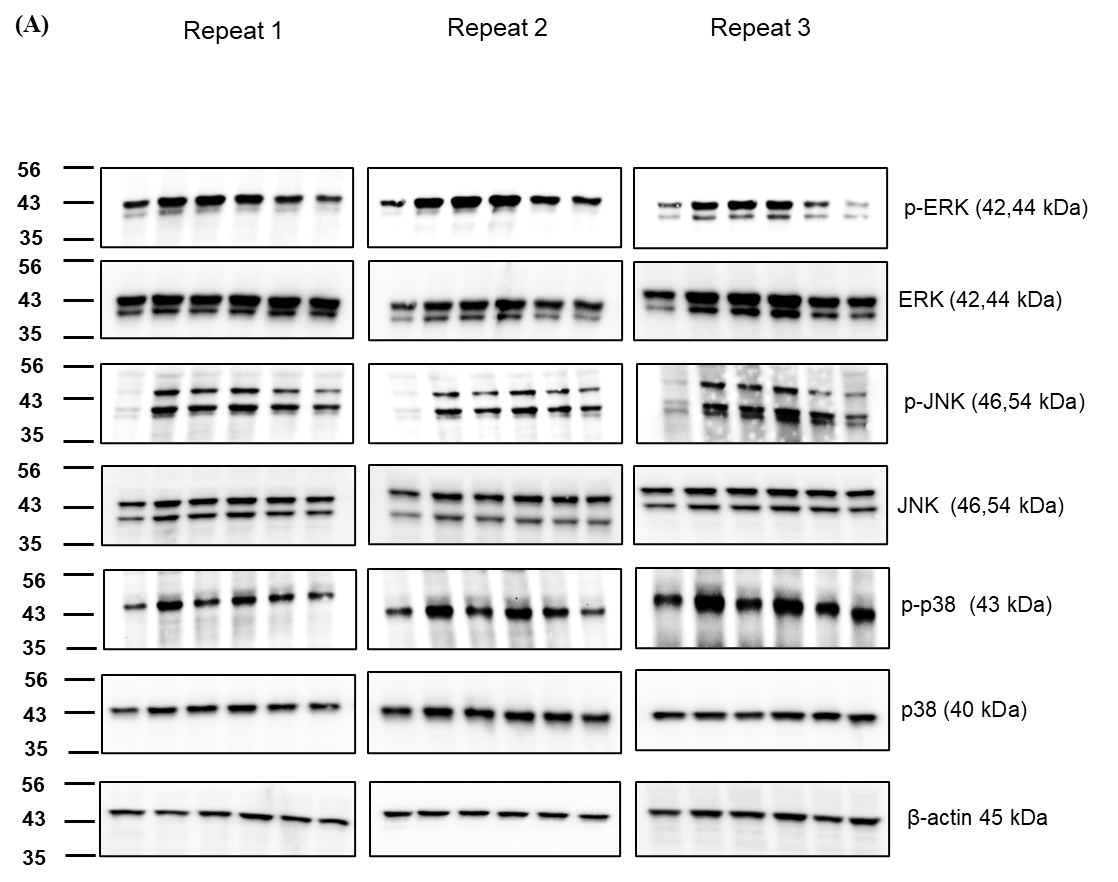


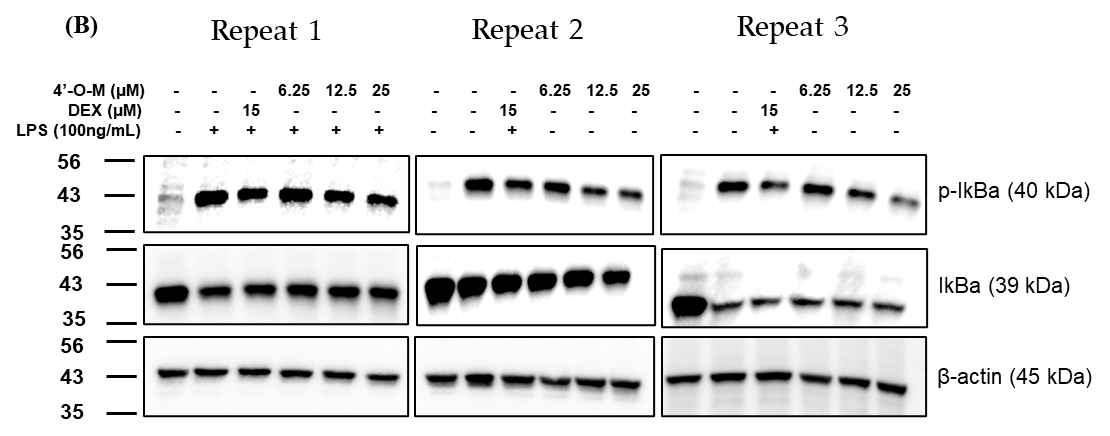


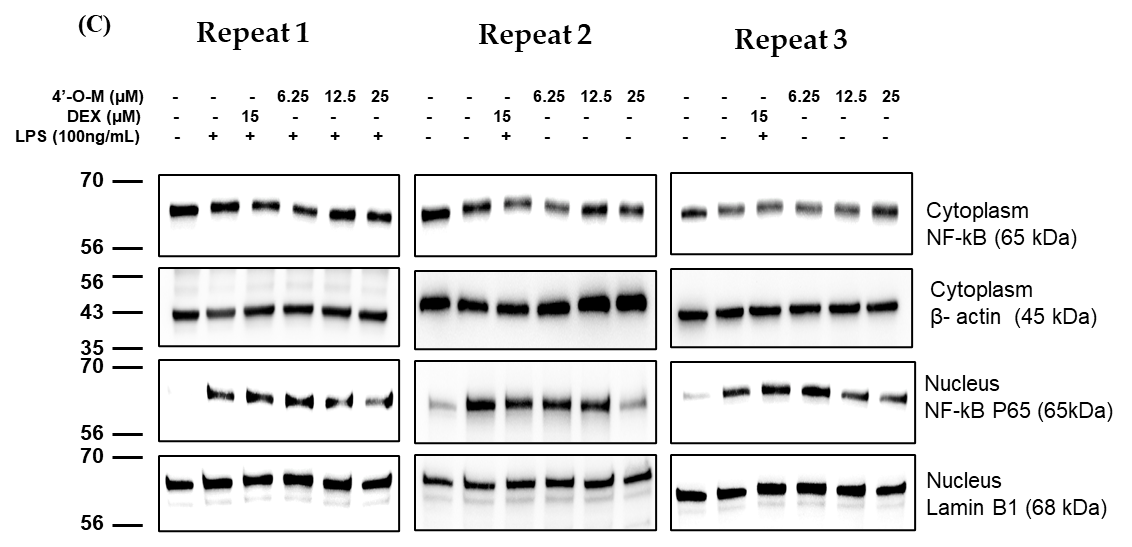


**Supplementary Figure 1.** Effects of 4′-O-M on ERK, JNK, p38 MAPK, IκBα, and NF-kB signaling in LPS-activated J774A.1 macrophages

Cells were pretreated with various concentrations (6.25, 12.5, and 25 μM) of 4′-O-M for 1 h and primed with LPS (100 ng/mL) for 2 h. (A) Expressions of MAPK-related proteins (B) IκBα-related proteins and (C) NF-kB-related proteins were analyzed by western blotting. Values are presented as means ± SD. Significant differences are indicated as *p < 0.05, **p < 0.01, and ***p < 0.001. Abbreviations: 4′-O-M, 4′-O-methylbroussochalcone B; MAPK, mitogen-activated protein kinase; NF-κB, Nuclear factor kappa B; ERK, extracellular signal-regulated kinase; JNK, c-Jun N-terminal kinase; IκBα, inhibitor of kappa B alpha; LPS, lipopolysaccharide; SD, standard deviation


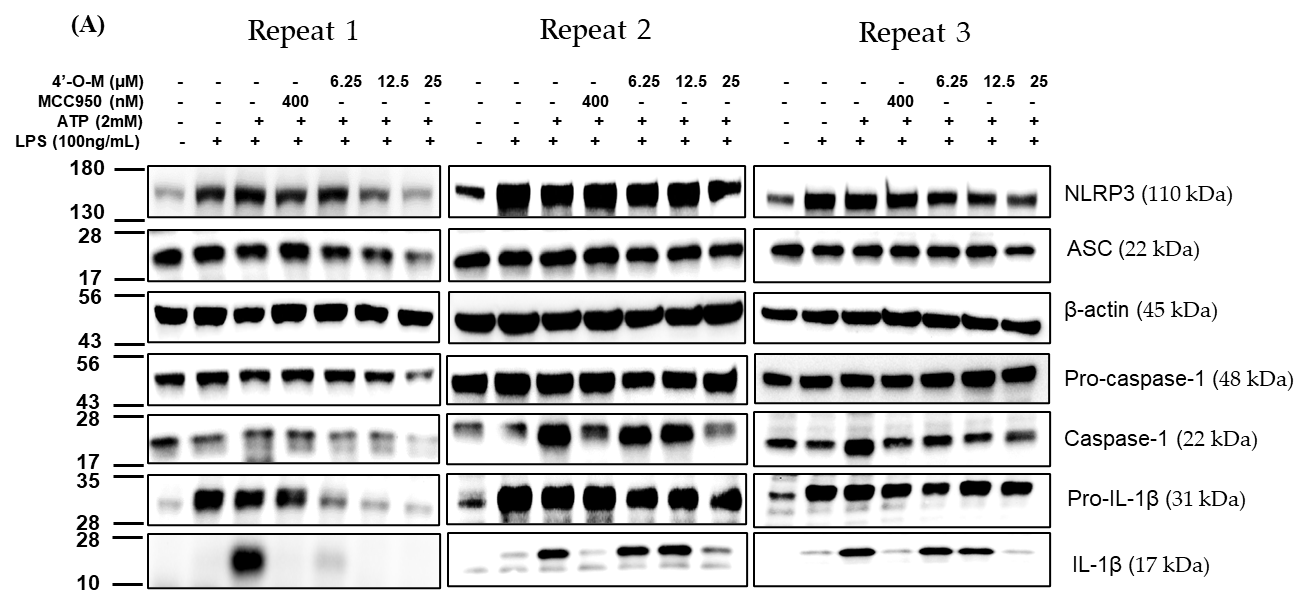


**Supplementary Figure 2.** 4’-O-M attenuates NLRP3 inflammasome activation by LPS/ATP-simulated J774A.1 macrophages

Cells were primed with LPS (100 ng/mL) for 6 h and treated with various concentrations of 4′-O-M (6.25, 12.5, and 25 μM), followed by stimulation with ATP (2 mM) for 30 min. (A) Expressions of the NLRP3-related proteins were analyzed by western blotting. (B) Cell culture supernatant was collected, and the expression of IL-1β, IL-18, and TNF-α was analyzed by ELISA. Values are presented as means ± SD. Significant differences are indicated as *p < 0.05, **p < 0.01, and ***p < 0.001. Abbreviations: 4′-O-M, 4′-O-methylbroussochalcone B; NLRP3, NOD-like receptor protein 3; LPS, lipopolysaccharide; ATP, adenosine triphosphate; IL, interleukin; TNF-α, tumor necrosis factor-α; ELISA, enzyme-linked immunosorbent assay; SD, standard deviation; ASC, apoptosis-associated speck-like protein containing a CARD


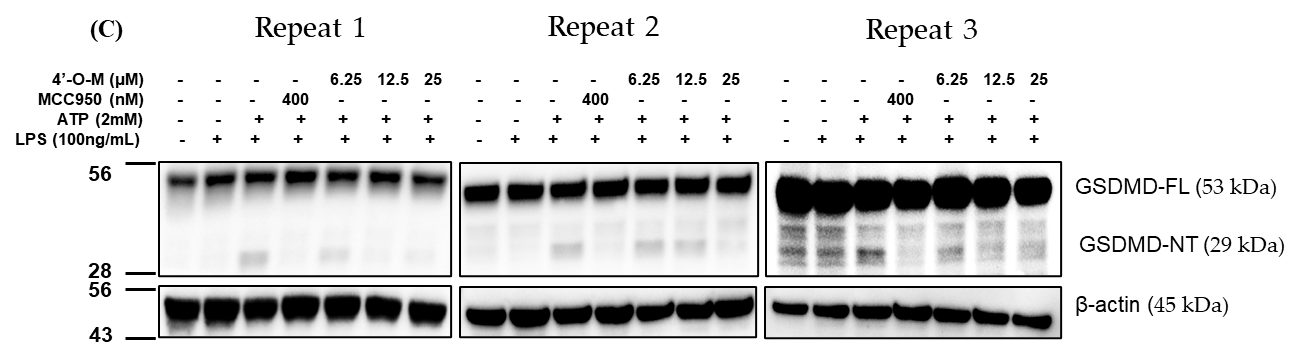


**Supplementary Figure 3.** 4′-O-M attenuates pyroptosis activation in LPS/ATP-simulated J774A.1 macrophages

Expressions of the pyroptosis-related proteins were analyzed by western blotting. Abbreviations: 4′-O-M, 4′-O-methylbroussochalcone B; LPS, lipopolysaccharide; ATP, adenosine triphosphate; GSDMD, gasdermin D

**
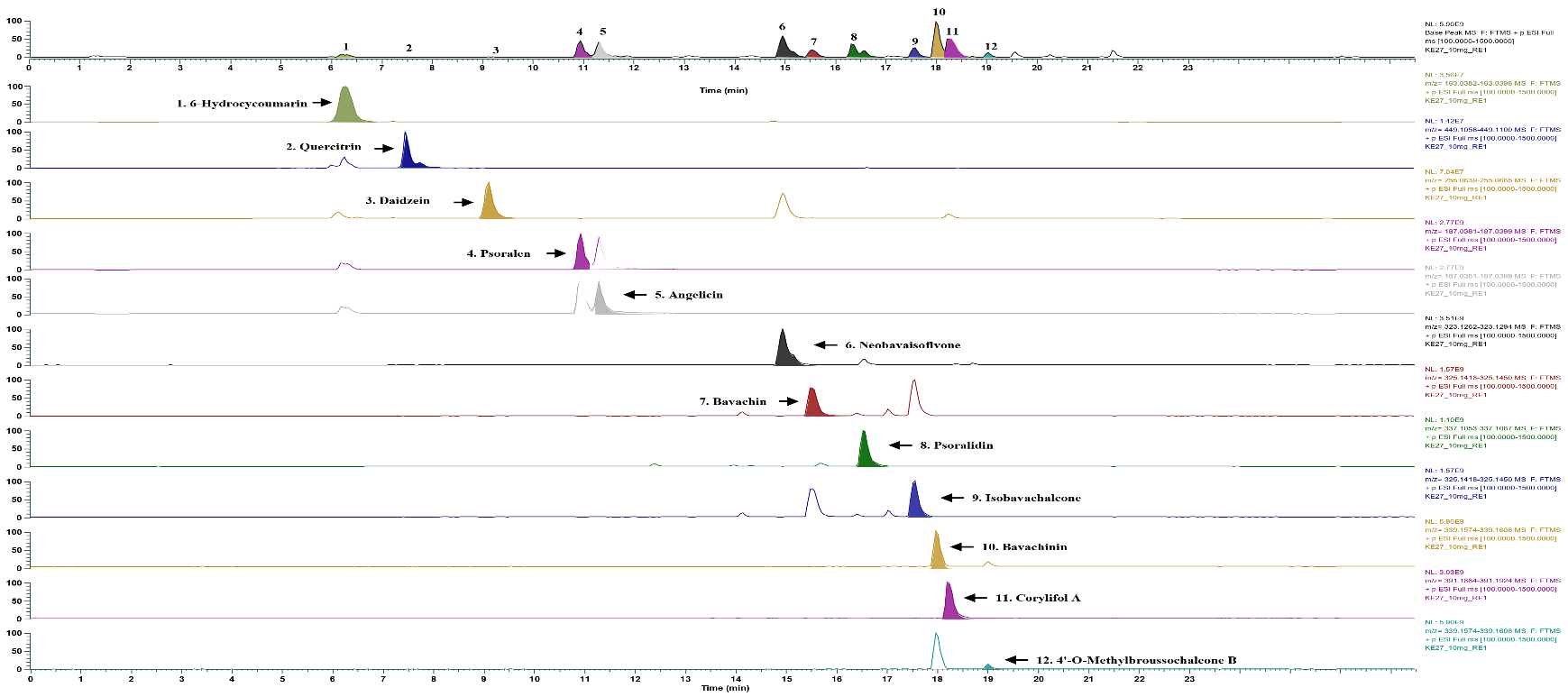
**

**Supplementary Figure 4.** Chromatograms from the LC-MS/MS of *Psoralea corylifolia* L. chalcones

Abbreviations: LC-MS/MS, Liquid Chromatography-Tandem Mass Spectrometry


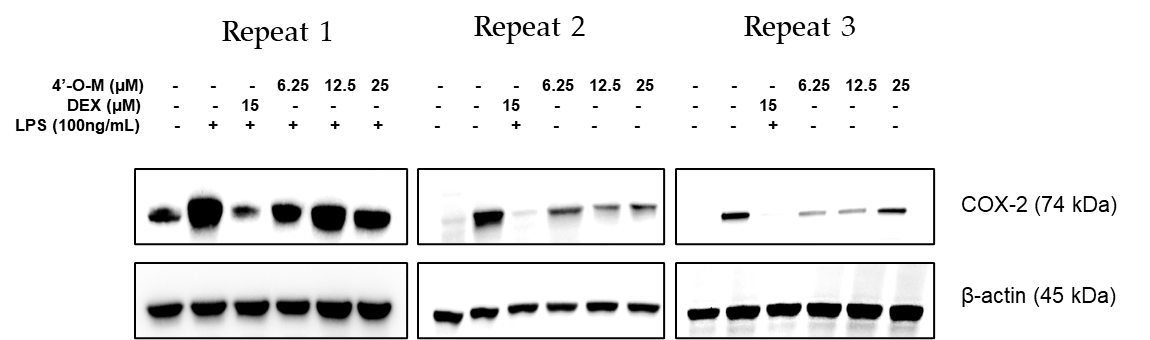


**Supplementary Figure 5.** Validation of the anti-inflammatory mechanism of 4′-O-M using western blot analysis in J774A.1 macrophages

Abbreviations: 4′-O-M, 4′-O-methylbroussochalcone B.

**Supplementary Figure 6.** Full-length Western blot membranes

Figure 2(A). Original Western blot images in triplicates

**
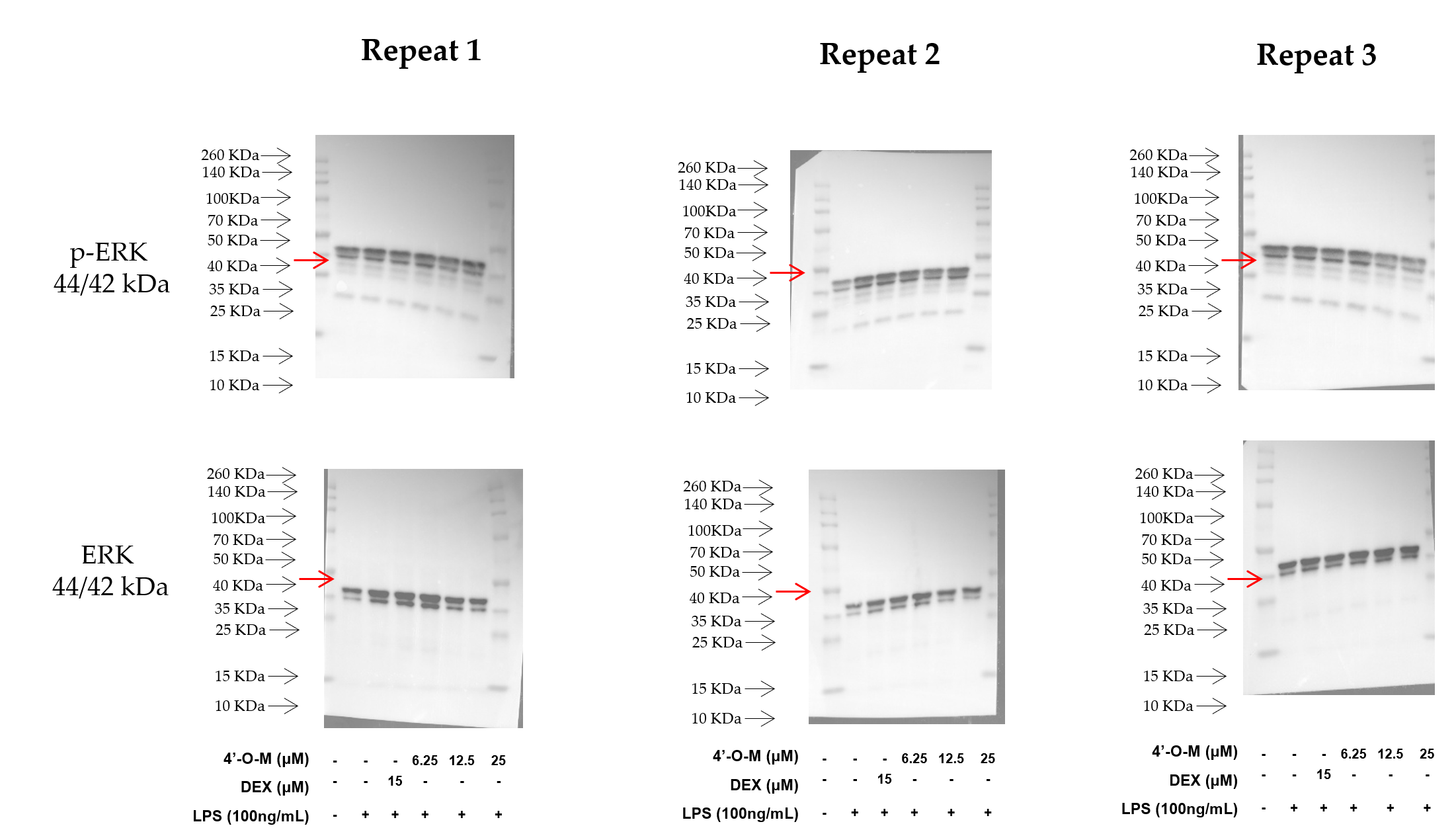
**


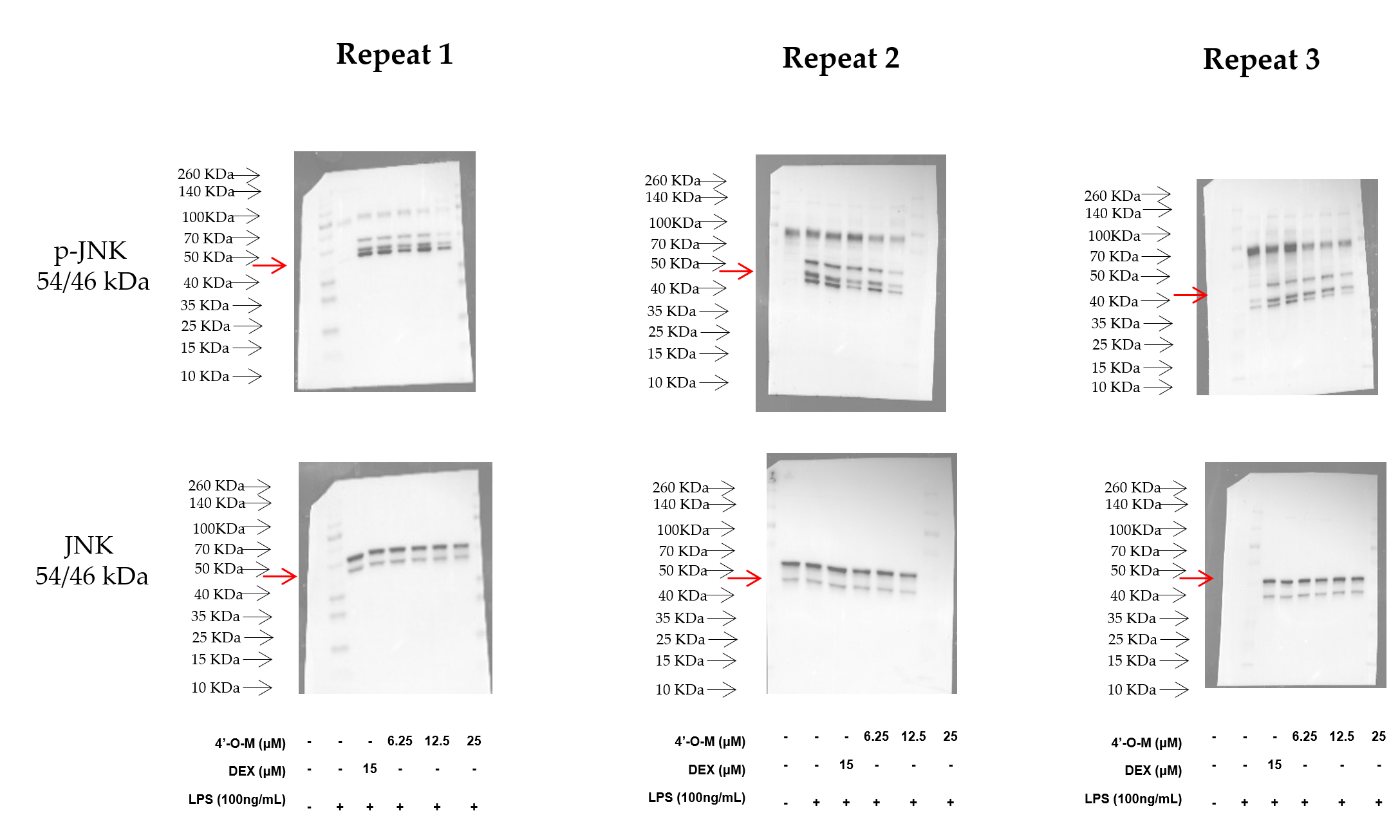


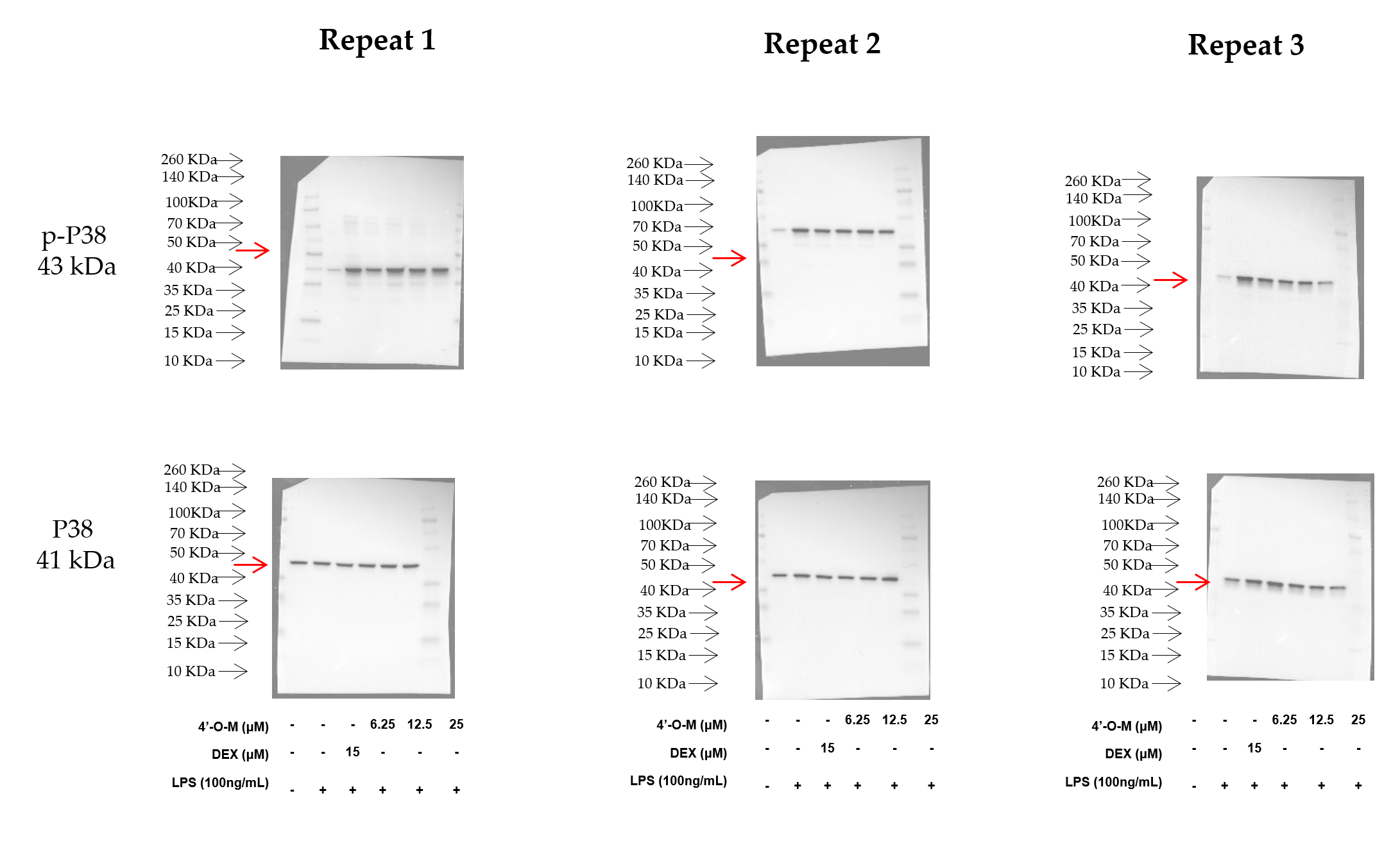


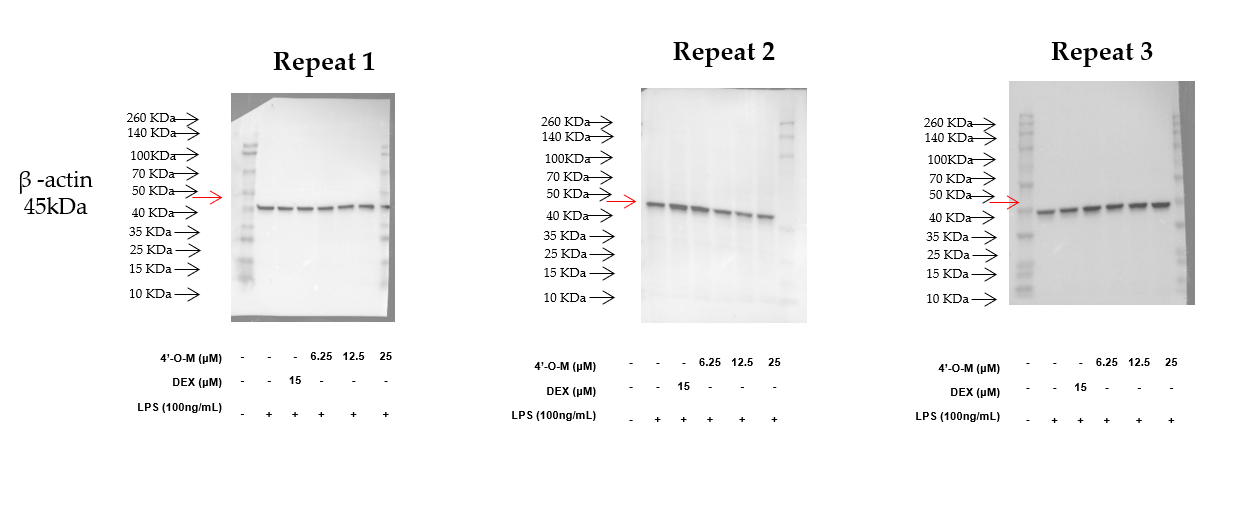


Figure 2(B). Original Western blot images in triplicates


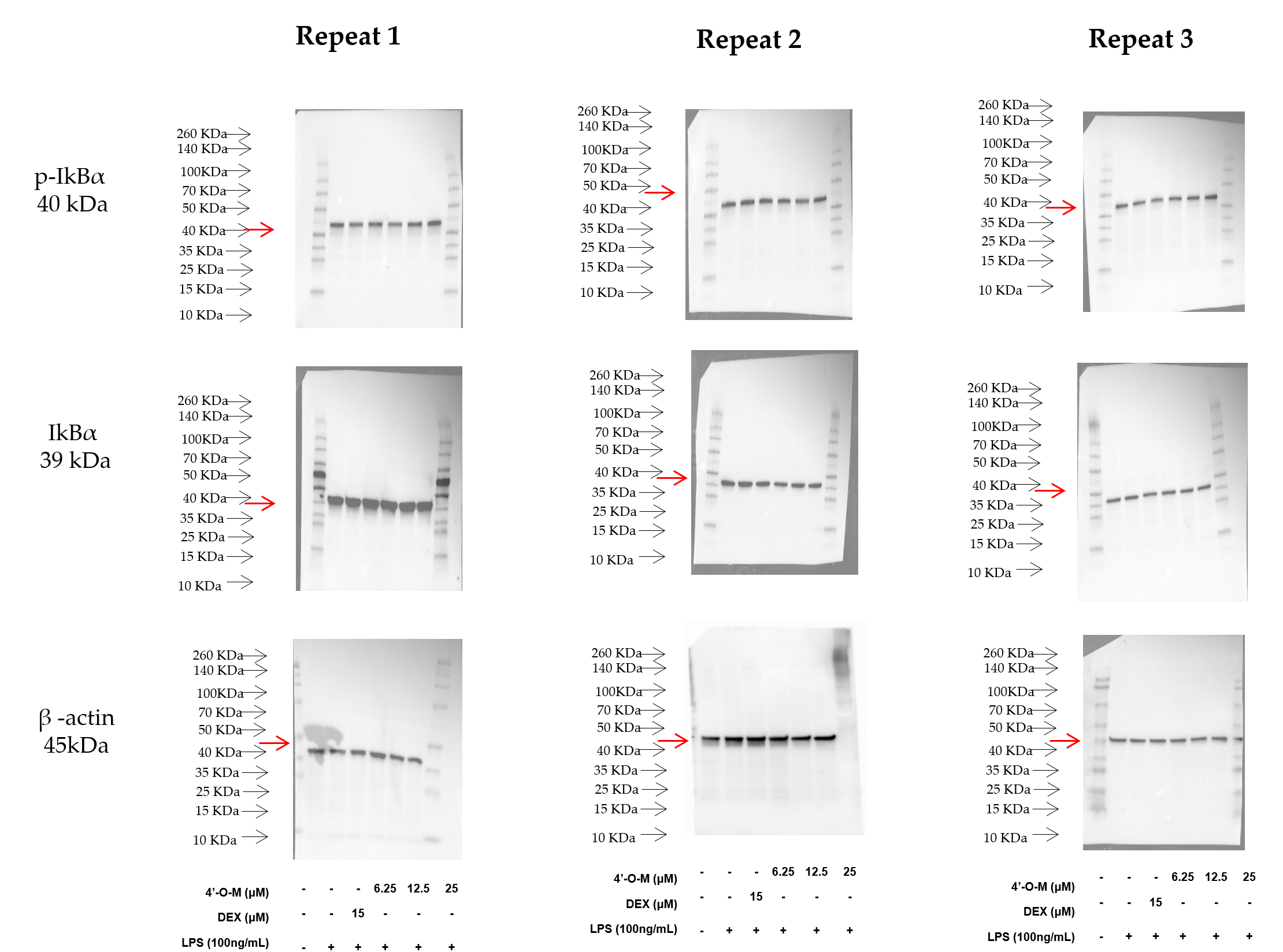


Figure 2(C). Original Western blot images in triplicates


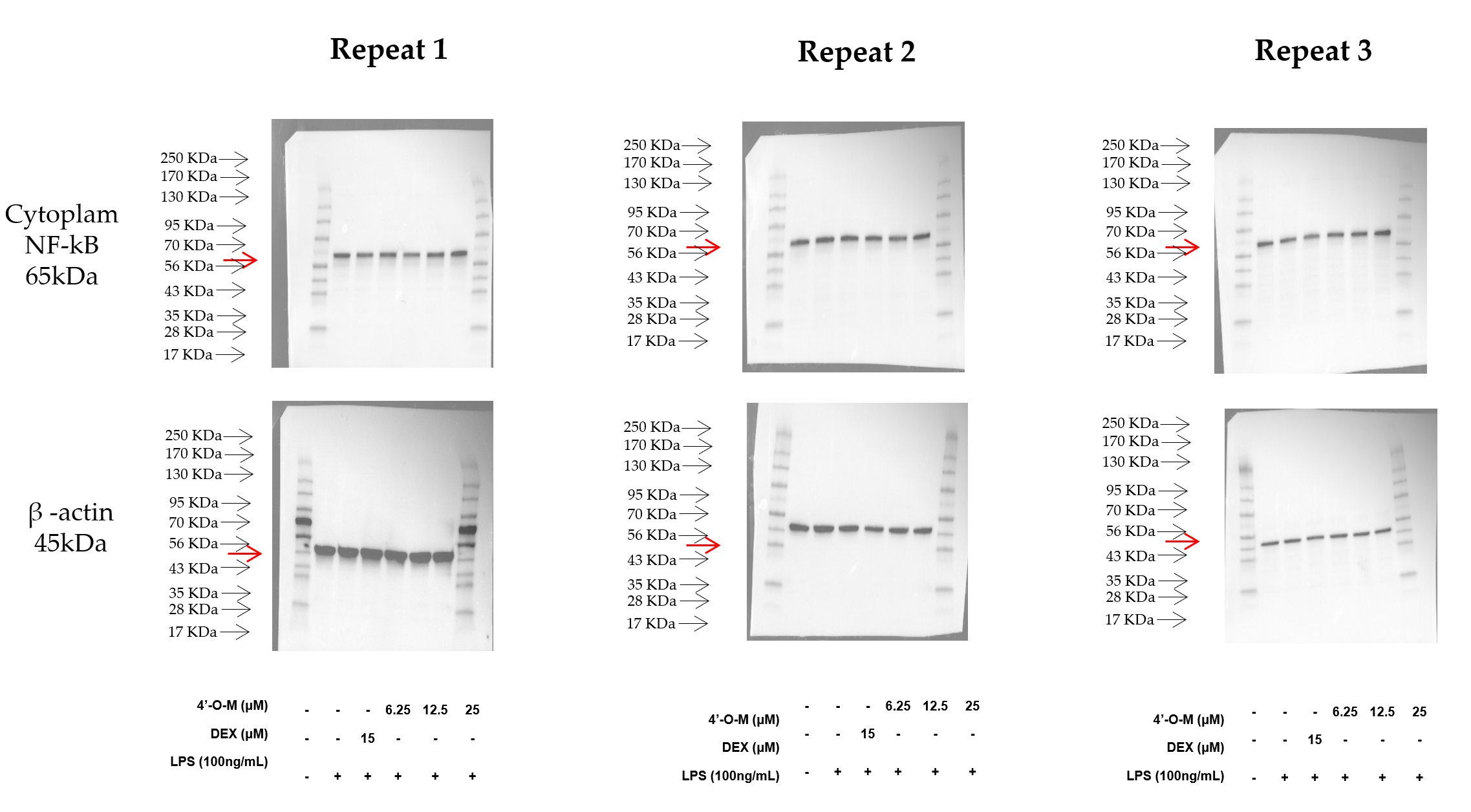


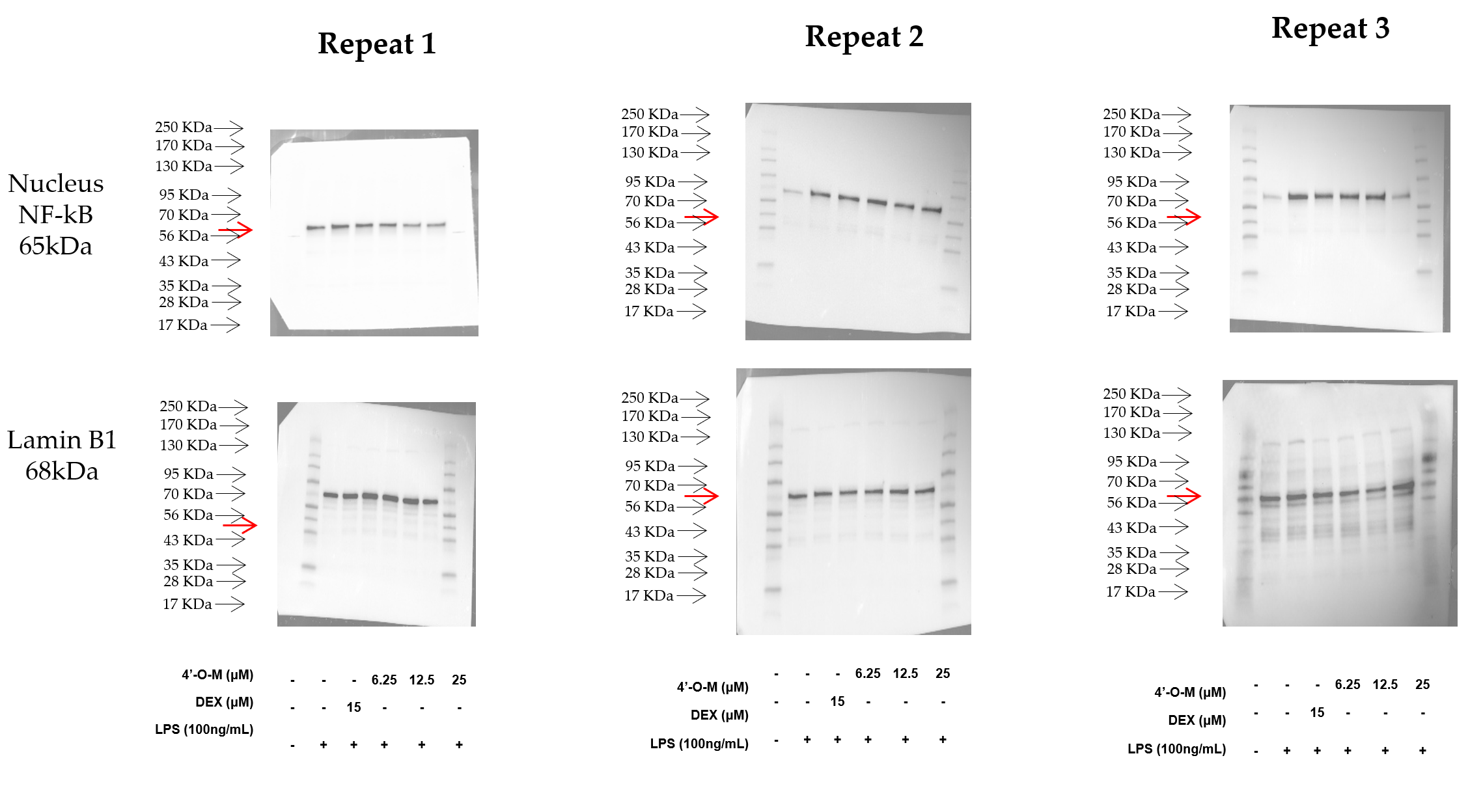


Figure 3 and 4. Original Western blot images in Repeat 1


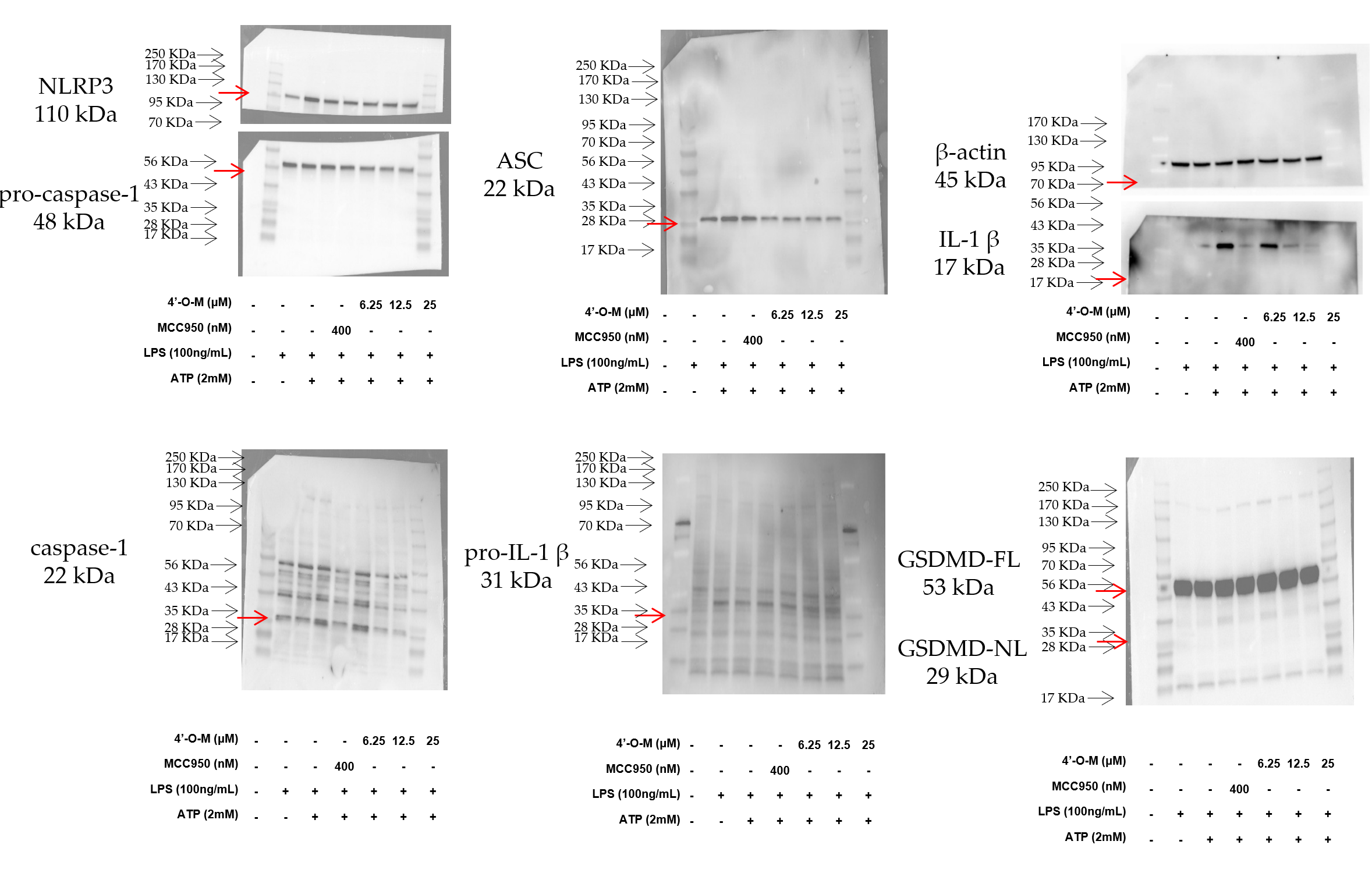


Figure 3 and 4. Original Western blot images in Repeat 2


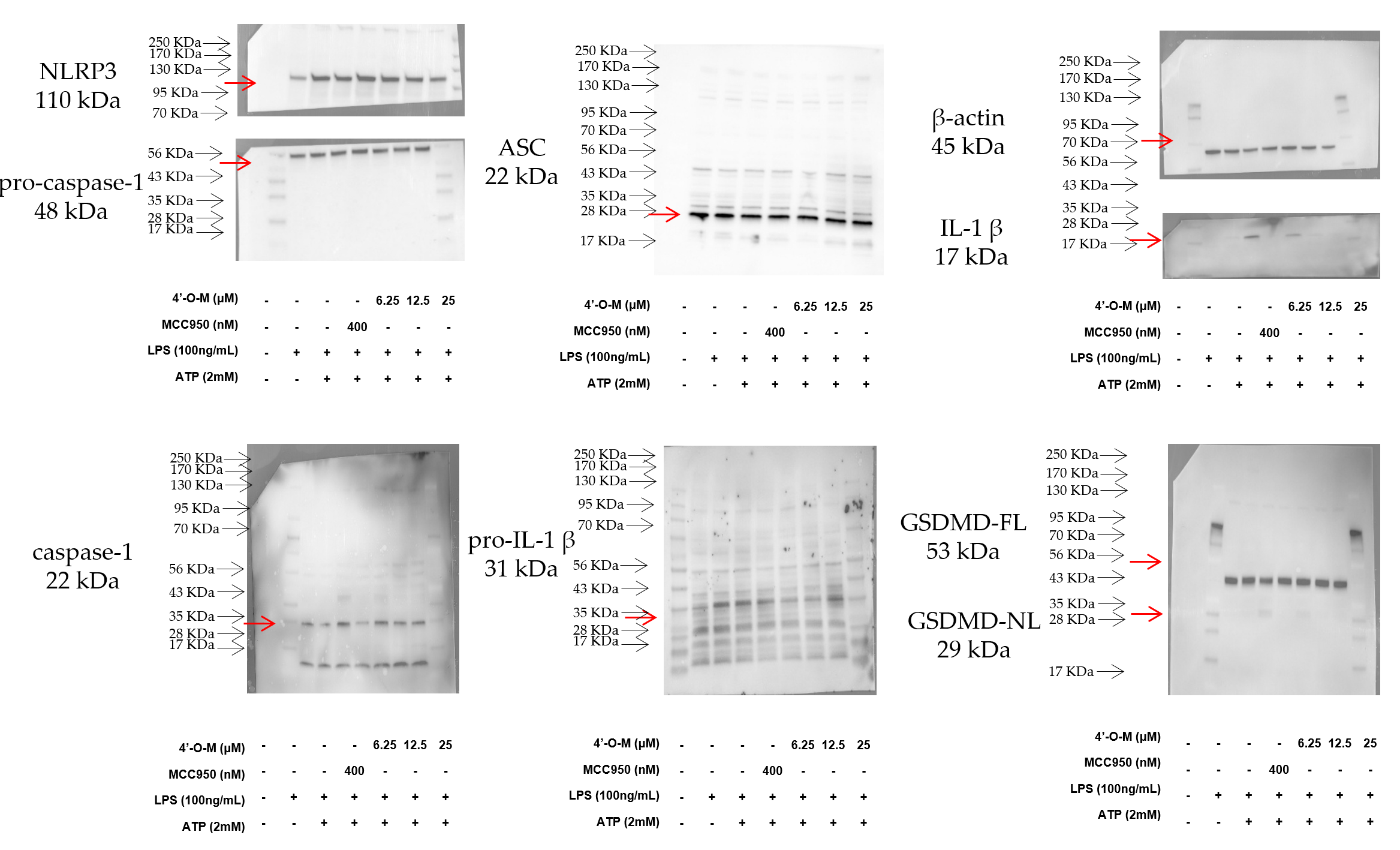


Figure 3 and 4. Original Western blot images in Repeat 3


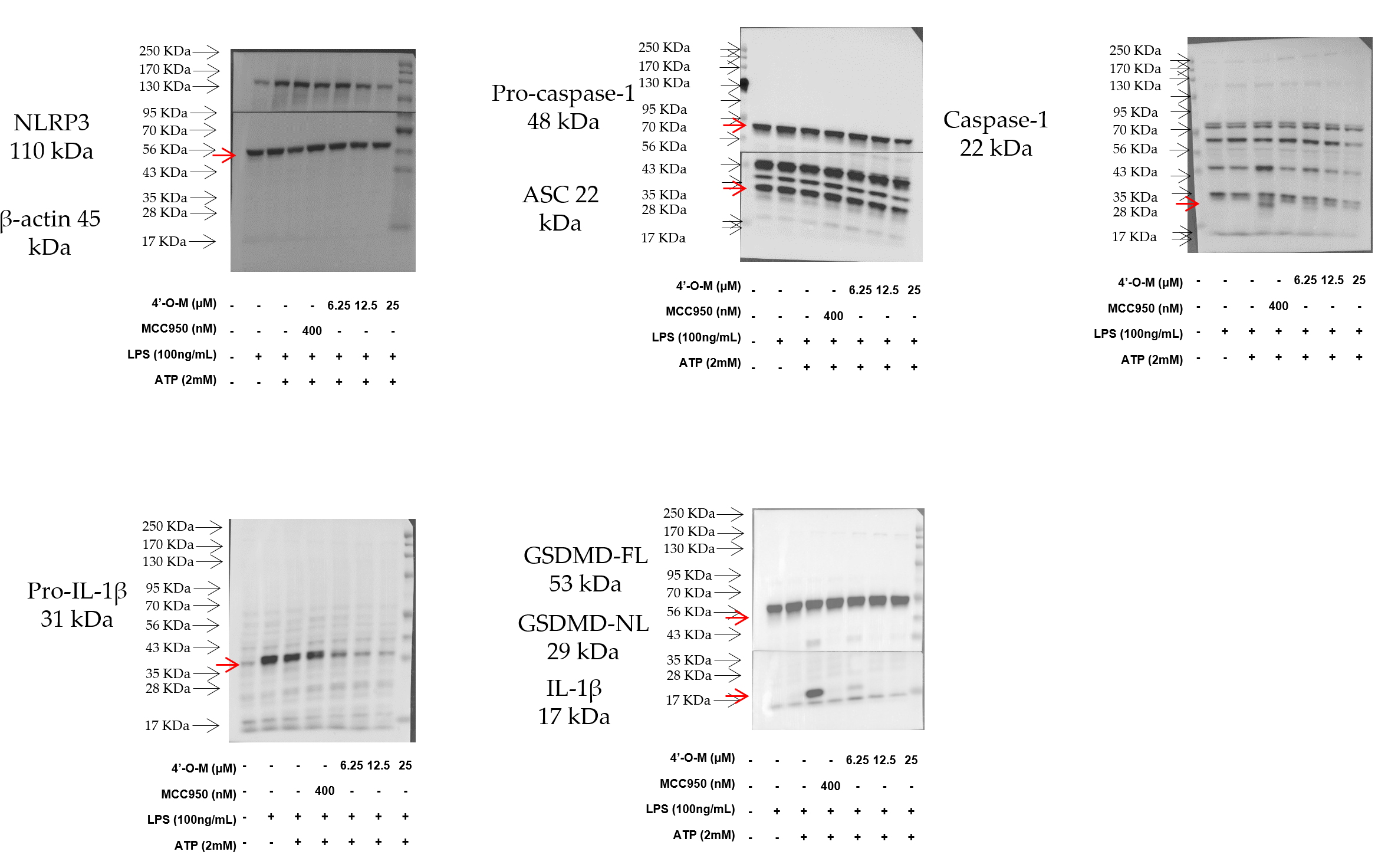

Supplement: Supplementary file 1 [file Supplementaryfile1.docx]
